# From cells to tissue: How cell scale heterogeneity impacts glioblastoma growth and treatment response

**DOI:** 10.1371/journal.pcbi.1007672

**Published:** 2020-02-26

**Authors:** Jill A. Gallaher, Susan C. Massey, Andrea Hawkins-Daarud, Sonal S. Noticewala, Russell C. Rockne, Sandra K. Johnston, Luis Gonzalez-Cuyar, Joseph Juliano, Orlando Gil, Kristin R. Swanson, Peter Canoll, Alexander R. A. Anderson

**Affiliations:** 1 Integrated Mathematical Oncology, Moffitt Cancer Center, Tampa, Florida, United States of America; 2 Precision NeuroTherapeutics Innovation Program, Mathematical NeuroOncology Lab, Mayo Clinic, Phoenix, Arizona, United States of America; 3 Department of Neurological Surgery, Mayo Clinic, Phoenix, Arizona, United States of America; 4 Department of Pathology and Cell Biology, Columbia University Medical Center, New York, New York, United States of America; 5 Department of Radiation Oncology, University of Texas MD Anderson Cancer Center, Houston, Texas, United States of America; 6 Division of Mathematical Oncology, City of Hope National Medical Center, Duarte, California, United States of America; 7 Department of Radiology, University of Washington, Seattle, Washington, United States of America; 8 Department of Pathology, University of Washington, Seattle, Washington, United States of America; 9 Keck School of Medicine, University of Southern California, Los Angeles, California, United States of America; 10 Department of Biology, Hunter College, City University of New York, New York, New York, United States of America; University of Southern California, UNITED STATES

## Abstract

Glioblastomas are aggressive primary brain tumors known for their inter- and intratumor heterogeneity. This disease is uniformly fatal, with intratumor heterogeneity the major reason for treatment failure and recurrence. Just like the nature vs nurture debate, heterogeneity can arise from intrinsic or environmental influences. Whilst it is impossible to clinically separate observed behavior of cells from their environmental context, using a mathematical framework combined with multiscale data gives us insight into the relative roles of variation from different sources. To better understand the implications of intratumor heterogeneity on therapeutic outcomes, we created a hybrid agent-based mathematical model that captures both the overall tumor kinetics and the individual cellular behavior. We track single cells as agents, cell density on a coarser scale, and growth factor diffusion and dynamics on a finer scale over time and space. Our model parameters were fit utilizing serial MRI imaging and cell tracking data from ex vivo tissue slices acquired from a growth-factor driven glioblastoma murine model. When fitting our model to serial imaging only, there was a spectrum of equally-good parameter fits corresponding to a wide range of phenotypic behaviors. When fitting our model using imaging and cell scale data, we determined that environmental heterogeneity alone is insufficient to match the single cell data, and intrinsic heterogeneity is required to fully capture the migration behavior. The wide spectrum of *in silico* tumors also had a wide variety of responses to an application of an anti-proliferative treatment. Recurrent tumors were generally less proliferative than pre-treatment tumors as measured via the model simulations and validated from human GBM patient histology. Further, we found that all tumors continued to grow with an anti-migratory treatment alone, but the anti-proliferative/anti-migratory combination generally showed improvement over an anti-proliferative treatment alone. Together our results emphasize the need to better understand the underlying phenotypes and tumor heterogeneity present in a tumor when designing therapeutic regimens.

## Author summary

Glioblastoma, the most common primary brain tumor, is an aggressive and difficult to treat cancer. A key reason is that the tumors can be very heterogeneous, consisting of many different mutants driving distinct cell behaviors. We believe that treatments for this disease could be significantly improved by understanding and quantifying the functional impact of heterogeneity within the tumor. From a clinical standpoint, the larger tissue-scale dynamics, like growth rate, can be informed from serial MRI imaging, while the cell-scale heterogeneity, can be informed by analysis of biopsies. In this work, we combined information from both scales using a mathematical framework and multiscale data from an animal model of glioblastoma. We found that a wide range of potential tumor compositions matched imaging data alone, as a result the model predicts a wide variety of responses to treatment. Using both imaging and cell-scale data narrowed the range of possible tumor compositions and better predicted responses to treatment. The mathematical model also predicted that while targeting migration alone did not slow tumor growth (in fact it drove a more proliferative tumor), an anti-proliferative/anti-migratory treatment combination improved treatment response.

## Introduction

Glioblastoma multiforme (GBM) is the most common and deadly form of brain cancer with a median survival rate of 12–15 months [1,2]. The extensive infiltration of single cells in and around important anatomical structures makes curative surgical resection practically impossible, and resistance to radiation and chemotherapeutic strategies often causes recurrence following an initial response. Magnetic resonance imaging (MRI) serves as the primary diagnostic viewpoint into the disease state and guides the subsequent treatment strategies that follow. However, it is often the case that patients with similar growth patterns determined with MRI will have different post-treatment kinetics. While patient data at smaller scales, such as histological and genetic profiling, is known to be generally prognostic, its connection to optimal therapeutics and clinical imaging remains an active area of research [3–8]. In this work, we investigate how phenotypic heterogeneity at the cell scale affects tumor growth and treatment response at the imaging scale by quantitatively matching multiscale data from an experimental rat model of GBM to a mechanistic computational model.

It is broadly acknowledged that GBMs exhibit genetic and phenotypic heterogeneity both spatially and temporally [9–13]. However, GBM progression is not just driven by cell autonomous genetic and epigenetic alterations but also from larger scale non cell autonomous interactions between cells and their environment [14–16]. Data is routinely collected in the clinic, but different scales are generally separated. Imaging gives us larger tissue scale information like size to quantify burden or density variations that can be used to define different environmental habitats [17–19]. Histology, single cell data, and genetic profiling can be used to view heterogeneity at the tissue and individual cell level, however, the measured heterogeneity at the cell scale does not directly lead to predictions in tumor growth and treatment response.

**Fig 1:**
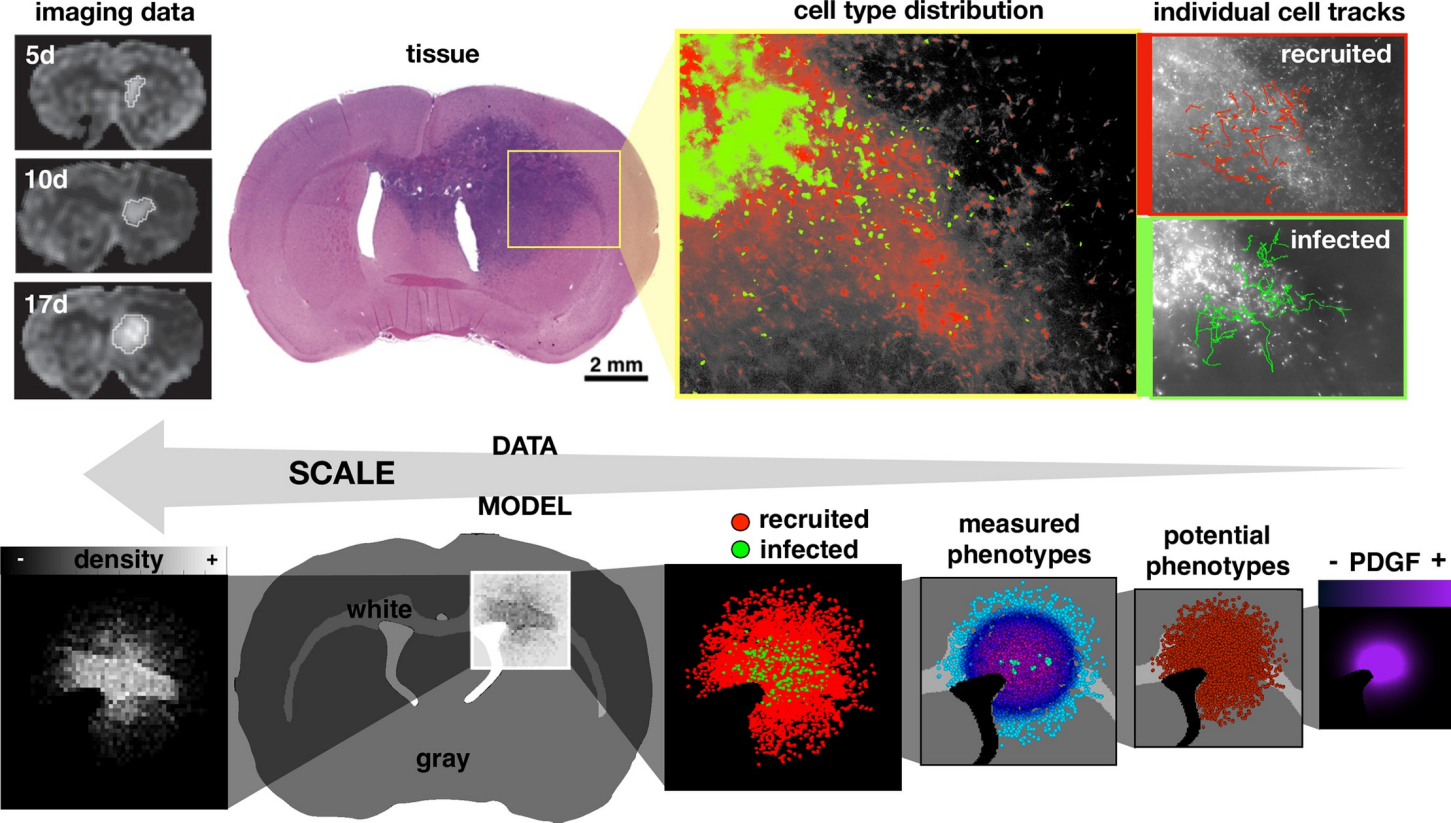
Coupling multiscale data to a multiscale mathematical model. Upper: data from rat experiments including imaging at 5, 10, and 17 days post injection, circumscribed and quantified from serial MRI images, tissue slice image, spatial distribution of infected (green) and recruited (red) cells, and individual cell tracks. Lower: the multiscale model represents the imaging as a spatial density map, considers the gray and white matter distribution in the rat brain tissue, and tracks cell types (infected and recruited), measured cell phenotypes (actual proliferation and migration), potential cell phenotypes (maximal proliferation and migration), and the PDGF concentration field.

Here we examine feedback between tumor and microenvironmental heterogeneity using a model that considers amplification of platelet-derived growth factor (PDGF). A transient PDGF signal is part of a normal injury repair response mechanism [20,21], as glial cells can be stimulated by PDGF to proliferate, migrate, and differentiate [20–22]. However, PDGF can be overexpressed in proneural GBM tumor cells to drive tumor growth and invasion [14,23–30]. The observed cellular phenotypic heterogeneity is a combination of intrinsic cellular variation and their response to the local environment. Whilst it is impossible to separate observed cell phenotypes from their environmental context *in vivo*, we can investigate this complex system using a mathematical framework coupled to multiscale data to get a more complete picture of the disease (Fig 1). In this work, we use MRI imaging data and *ex vivo* time lapse imaging of fluorescently tagged cells in tissue slices (Fig 1 upper) to parameterize a mechanistic hybrid agent-based model (Fig 1 lower).

Mathematical models have been developed to study many facets of GBM growth and response to treatment [5,22,31–43]. There have been numerous papers published by Swanson *et al* demonstrating the clinical use of a relatively simple partial differential equation model based on net rates of proliferation and invasion. To date they have used their models to predict therapeutic benefits from surgery and radiation [44–47], IDH1 mutation status [48], and implications of growth kinetics during PDGF-driven tumor progression [33,34]. However, the continuum nature of this model means it cannot capture intercellular heterogeneity which may impact long-term post treatment behavior. Here, we consider intratumor heterogeneity in proliferation and migration rates from inheritable phenotypes at the cell scale and from the microenvironment. The multiscale nature of our hybrid model enables us to tune our parameters with both imaging and cell-tracking data, thus allowing us to predict a host of tumor behaviors from size to composition to individual cell responses to therapy. This could be key to understanding treatment response as single cells can cause relapse or treatment failure.

In the following sections, we introduce the experimental model by Assanah *et al* of PDGF-driven GBM in which single cells were tracked. We then present a hybrid agent-based mathematical model which is able to capture the spatial and temporal heterogeneity of single cells. Using this model, we first identify the sets of parameters with which our model is able to recapitulate the observed tumor size dynamics from the data. We then identify the sets of parameters that fit smaller scale metrics from the data, such as the observed distribution of individual cell velocities. We investigate how the fully parametrized model with both intrinsic and environmental heterogeneity compares to a case where all cells are intrinsically homogeneous within a spatially heterogeneous environment, and finally, we show how anti-proliferative and anti-migratory drugs affect outcomes and modulate heterogeneity within the tumor cell population.

## Methods

### Ethics statement

The University of Washington, Seattle approved the study to use human tissue. The initial IRB approval number was HSD: 43264, and the current approval number is STUDY00002352, due to a change in the IRB system. Form of consent was written. There were instances where consent was waived where patients were deceased (roll-over from another IRB approved study) or lost-to-follow-up (from another IRB approved study).

### Rat model and *ex vivo* multiscale data analysis

The experimental rat model enabled the tracking of both cells that were infected with the PDGF-over-expressing retrovirus, tagged with green fluorescence protein (GFP), and normal recruitable progenitor cells, tagged with dsRed. At 2 and 10 days post infection, brains were excised and cut into 300μm thick slices, and positions of labeled cells and their progeny were tracked by hand every 3 minutes from time-lapse tracking. For more details on the experimental model, see [14]. A total of 751 cells were tracked (152 infected and 188 recruited at 2d and 203 infected and 208 recruited at 10d) in the tissue slices (2 slices at 2d and 4 at 10d) over time. Proliferation rate was calculated by dividing the number of proliferation events over the time period by the total number of cells at the beginning of the observation period and the total observation time in hours. For each cell we calculated a cell speed by the total distance traveled over the total time spent moving. The persistence times for moving and stopping, and the turning angles were also calculated (see S1 Methods).

### Hybrid off-lattice agent-based mathematical model

Our hybrid model consists of tumor cells, represented as off-lattice agents, and a PDGF distribution, represented as a continuous field. We used off-lattice agents to allow single cells to migrate without the confines of a grid structure, but used a larger scale square lattice to track the cell density matrix, which we used to check if the local carrying capacity was reached. A smaller hexagonal lattice was used to track PDGF dynamics and define the brain tissue in terms of white and gray matter.

#### Model initialization and flow

**Fig 2:**
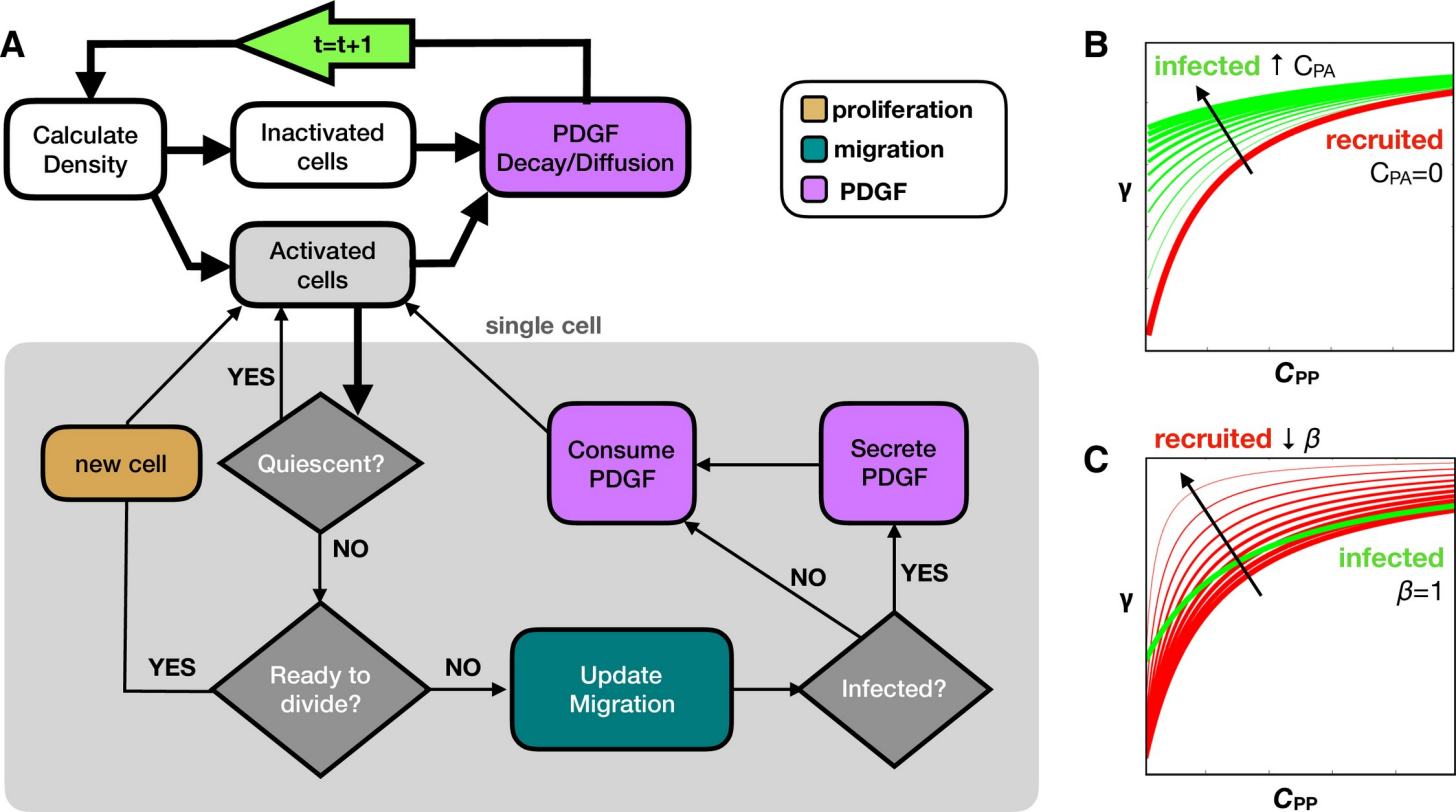
Computational model overview. A) Flow chart shows key decision points in the model. Tissue processes are connected with thick black lines, while the cell loop for single cell processes are contained within the gray box and connected with thin black lines. At the start of each time step (green arrow), we calculate the density and find the activated and inactivated subsets of cells. All activated cells are checked for quiescence, division, migration, and PDGF interactions as shown. Then PDGF decay and diffusion occurs before moving onto the next time step. The infected and recruited cells respond differently to PDGF due to B) an autocrine stimulation for infected cells (*C*_PA_ in Eq 2) and C) a decreased activation barrier for recruited cells (*β* in Eq 2). Increasing *C*_PA_ shifts the response upward at low *C*_PP._ Decreasing *β* increases the slope to achieve high response at lower *C*_PP_, while still inactive at *C*_PP_ = 0.

We define the white and gray matter using a section from an 80 day old male Sprague Dawley rat [49–51] using the Scalable Brain Atlas [52]. We selected a coronal slice near the bregma to get a representative 2D brain field involving the corpus callosum (Fig 1 bottom). For simplicity, any anatomical tissue feature that was not white matter was rendered as gray matter. The final array defines an 833x573 pixel domain corresponding to a scaled brain size of roughly 14.5x10.0 mm. There is an initial injection of 100 infected cells, which are labeled green and produce PDGF, and 100 recruited progenitor cells, which are labeled red and do not produce PDGF. In addition, a field of inactive but recruitable progenitor cells are randomly initialized throughout the brain matter at variable density around 2%, corresponding to the oligodendrocyte progenitor population [53,54]. There is also an initial bolus of PDGF, representing an injury response caused by the injection [14]. The flowchart in Fig 2A details the major decisions at each time point about division (orange), migration (teal), and PDGF (purple). All cells are assumed to be 25 μm in diameter.

#### Calculate cell density matrix

We define a coarse square mesh (100μm x 100μm) to check the local cell density. Each cell is assigned a closest neighborhood, which has a carrying capacity of κ in gray matter and 2κ/3 in white matter. We also check recruitable cell activation at this step, as the field of recruitable cells remain inactive unless the local PDGF is greater than 5x10^-4^ng/mL. Only activated cells are termed “recruited” and go through the cell loop.

### Cell loop

1. Proliferation and quiescence: A cell’s intermitotic time acts as a timer for division, counting down at each time step until the end of the cycle. At that point, a new cell is created at a random angle one radius away from the parent cell. However, if the number of cells in the neighborhood mesh point exceed the carrying capacity, then it is deemed quiescent, and it does not move forward in its cell cycle and does not divide. If subsequently there is enough room to divide, the cell reenters the cell cycle where it left off. The newly divided cell inherits the same proliferation rate and migration speed as its parental cell.

2. Migration: Glioma cells migrate in a stop and go fashion [55]. We randomly choose a migration status (stop or go), and sample from the distribution of persistence times. For a given persistence τ, a stopped cell will remain stopped and a moving cell will continue to move at the current angle and velocity. After τ time, the cell resets its migration status (stop or move), resamples τ from the data, and finds a new moving angle. In gray matter, cells do a random walk for τ sampling from a uniform distribution of turning angles, and in white matter, cells do a persistent random walk for 1.5τ sampling from a normal distribution centered around 0 with a standard deviation θ. A cell is not allowed to move into empty space, such as past the edges of the brain or within the ventricles. If a cell lands in this space, it has 10 attempts to find a suitable spot at other random angles. If unsuccessful, the distance moved is increased by a cell diameter, and the angle search is repeated for distances of up to 3 diameters away from the original location. If an empty space is not found, the cell remains in the original location (however, in our testing, a new location was always found before this constraint was satisfied). If the cell is set to move into a space that is already at carrying capacity, then it can move there only if it is less dense than the original space. Otherwise, it remains in place. This allows the density of cells to slightly surpass the carrying capacity but prevents much movement when above or near the carrying capacity.

3. Response to PDGF: PDGF can stimulate glial cells to proliferate and migrate by autocrine and paracrine signals [20,56]. Since we are interested in phenotypic heterogeneity in the tumor with regards to proliferation and migration, we need to separate the influence of the environmental PDGF, which can change depending on location, from the potential phenotype, which is inherited. To achieve this, we model the cells such that their observed phenotype for proliferation rate *p* and migration speed *m* is a product of the response to PDGF in the environment and some internal, inheritable upper limit:

$$\left(\begin{array}{c} p\\ m \end{array}\right)=\left(\begin{array}{c} p_{\mathrm{pot}}\gamma \left(C_{P}\right)\\ m_{\mathrm{pot}}\gamma \left(C_{P}\right) \end{array}\right),$$ (1)

where *p*_*pot*_ is the maximal potential proliferation rate, and *m*_*pot*_ is the maximal potential migration rate. The function *γ(C*_P_*)* represents how the concentration of PDGF *C*_P_ modulates the proliferation and migration, which ultimately takes a value from 0–1, so that *as C*_P_ becomes saturated proliferation and migration reach their maximum potential values *(*i.e. *γ(C*_P_*)→*1, so that *p→p*_*pot*_ and *m→m*_*pot*_). The exact functional relationship of *C*_P_ on *p* and *m* is not well established, but a Hill function response in compatible with the data [22,57]:

$$\gamma (C_{P})=\frac{C_{P}}{C_{P}+K}=\left\{ \begin{array}{c} \frac{C_{\mathrm{PA}}+C_{\mathrm{PP}}}{C_{\mathrm{PA}}+C_{\mathrm{PP}}+K}\;for\;infected\;cells\\ \frac{C_{\mathrm{PP}}}{C_{\mathrm{PP}}+\beta K}\;for\;recruited\;cells \end{array}\right.,$$ (2)

where *C*_PA_ is the PDGF contributing to the autocrine stimulation, *C*_PP_ is the PDGF contributing to the paracrine stimulation, K is the concentration at which the response is half maximum, and *β* modifies the activation barrier of recruited cells to PDGF stimulation. While all cells can respond to PDGF produced by the infected cells that diffuses throughout the surrounding environment *C*_PP_, only the infected cells have an autocrine effect, due to a portion of the PDGF *C*_PA_ that stays within and stimulates the infected cells. The recruited cells are also assumed to have a lowered activation barrier to *C*_PP_. We incorporate this into the equation by lowering the concentration at which the response is half maximum (by modifying *K* by *β*∊(0.1,1)), which causes recruited cells to gain a larger response from *C*_PP_ than infected cells while still being inactive when *C*_PP_ = 0. The effects of changing these values are shown in Fig 2C and 2D. Because there are a large number of inactive recruitable cells in the environment, we cut off any activity from these cells in areas with *C*_PP_<5x10^-4^ng/mL, which corresponds to an upper bound of 0.1% for the response function (*γ*(*C*_P_)< = 0.001) with the given parameter ranges. This cutoff reduces the computational expense from behavior that is essentially negligible.

4. PDGF secretion and consumption: Only infected cells secrete PDGF and all cells consume PDGF into or from the nearest hexagonal grid point. If there is less local PDGF than the amount to be consumed for a cell during the time step, all PDGF in the grid point will be consumed.

#### PDGF dynamics

A fine hexagonal mesh with the same radius of a cell (12.5 μm) is utilized for the PDGF dynamics. Following the cell loop, the whole PDGF field is subject to decay and then diffusion (further details in S2 Methods).

## Results

### Cell behavior in *ex-vivo* assay is influenced by multifaceted factors

**Fig 3:**
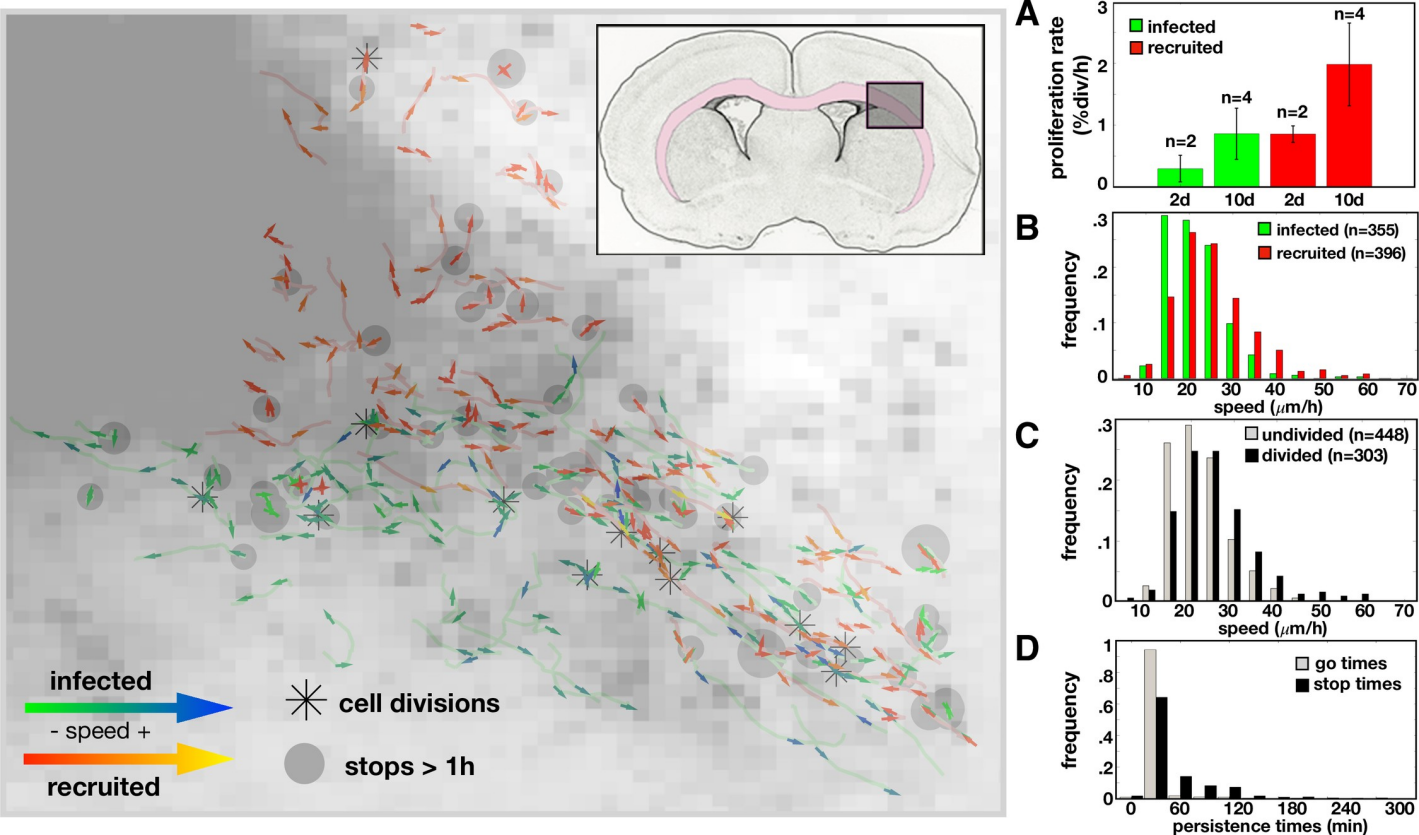
Data from the rat experiment. Left: Single cell trajectories at 2 days post infection overlaid on the cell density map. The insert shows the region of interest within the rat brain where the pink highlights the white matter. An asterisk marks where a cell division occurred. Each track contains an arrow for the first and last half of the track showing the average direction and speed over that time period. The arrows for the infected cells are green for lower speeds and blue for higher speeds. The arrows for recruited cells are red for lower speeds and yellow for higher speeds. Gray dots mark where a cell has stopped longer than 1 hour with the size proportional to the stop time. Right: Metrics derived from data. A) Proliferation rates (in % divisions per hour) at day 2 and 10 for infected and recruited cells (for n trials indicated). Speed distributions (calculated as distance traveled over time travelling in *μ*m/h) for B) infected vs recruited cells (mean speeds: 21.7*μ*m/h vs. 25.0*μ*m/h, respectively) and for C) undivided vs divided (mean speeds: 25.3*μ*m/h vs. 22.1*μ*m/h, respectively). D) Time spent during periods of movement or stopping for all cells (42.6min vs. 70.1min, respectively).

In a series of experiments by Assanah *et al*, it was shown that infecting resident glial progenitor cells with a retrovirus engineered to overexpress PDGF in the rat brain can induce a massive overgrowth of cells with histologic features similar to GBM [14,25]. The tumors grow rapidly and are composed of a mixture of retrovirus infected and uninfected/recruited progenitor cells [14]. Specifically, the tumor diameters at 5, 10, and 17 days post infection were 1.7, 2.4, and 3.2 mm, respectively, which were determined previously from MRI images in Massey *et al* [33]. At 17d, progenitors made up 80% of all labeled cells in the tissue section [14]. Single cell trajectories from the infected (green) and recruited (red) cells at 2d were tracked and are displayed in the spatial plot of Fig 3 along with births, stops, and speeds along the tracks. Cells were mainly measured near the edge of the tumor where the density was lower, so they could be distinguished from their neighbors. We found that there was a high degree of phenotypic heterogeneity amongst cells, some of which may be due to environmental influences. This is outlined below.

#### Phenotypic metrics

About half of the cells divided over the 25h track recording at 10d, and no cell during this time period divided twice, therefore the proliferation rate was quantified as a bulk population metric defined by the percentage of cells that divided over time (Fig 3A). This proliferation metric increased from 2d to 10d and was slightly higher for recruited cells (in agreement with the analysis in [14]). There was large variation in the trajectories of the cells (S1A Fig), and different metrics highlight specific features of the migration behavior. The average speed was shown to be slightly higher for recruited cells compared to infected cells by the mean squared distance (S1B Fig). Comparing the distributions of mean distance travelled over the time moved for each cell, we find significant differences in the histograms for recruited vs infected (Fig 3B) and cells that had divided, which includes the mother cell prior to division and daughter cells after division, vs those that did not divide (Fig 3C), whilst the difference between day 2 and 10 was less pronounced (S1C Fig). If we separate the trajectories into directional runs separated by turns and stops (as described in S1 Methods), we find that the distribution skews to include even more slower run speeds and a longer tail with some cells moving up to 100*μ*m/h (S1D Fig). The cells moved and stopped often, but we found that on average, cell stop times were longer than moving times (Fig 3D). They generally moved in the same direction, but occasionally made large turns (S1E Fig). S1 Table shows the migration and proliferation metrics for this data from 2d and 10d.

#### Possible environmental influences

The contrast-enhancing core of the tumor contained mostly viable and actively migrating and proliferating cells too dense to accurately track cells. Therefore, single cell trajectories were taken from the tumor edge. From these single cell trajectories, we were able to observe where cells moved, turned, divided, and stopped for long periods of time. From the early time points (Fig 3, left), cells appeared to move generally along the diagonal, between the top-left and the bottom-right of the region, which corresponds roughly to the white matter region highlighted in pink in the insert. There was also faster and more directional movement along the white matter tract while the denser areas of the tumor core and the outer gray matter areas generally had shorter, less directional paths. The long stops and the cell divisions were scattered throughout the tissue and didn’t significantly correlate to the local density or each other.

### *In silico* tumors with similar growth dynamics may have widely different compositions

Using the multiscale data from the experimental model: tumor size over time, a count of cell types, the percentage of proliferating cells in the population over time, and migration behavior tracked from single cells (S1 Table), we calculate similar metrics in the *in silico* tumors (see S3 Methods). We focused on a set of 16 uncertain parameter values with reasonably-defined search ranges (Table 1) and used a hybrid genetic algorithm-random sampling technique [58] to find parameter sets that fit the model to the time course of tumor sizes from the data at 5d, 10d, and 17d to within 10% error (S2A and S2B Fig).

**Table 1:** List of all variable trait ranges in the mathematical model. They are categorized into tissue-related, PDGF-related environmental effects, and cell specific values, such as response to PDGF or heterogeneity in proliferation and migration traits.

	PARAMETER	SYMBOL	RANGE (UNITS)	SOURCES
tissue	Recruitable cell density	_*ρR*_	0.1–5 (%)	[53,54]
	directionality deviation in white matter	*σ*_*θ*_	0–45 (degrees)	-
PDGF	Initial PDGF	*p*_*0*_	100–600 (ng/mL)	estimated
	Diffusion coefficient for PDGF	*D*_*p*_	1-1000(x10^-6^ cm^2^/day)	estimated
	PDGF decay rate	*r*_*d*_	0–0.500 (ng/mL.day)	estimated
	PDGF secretion rate	*r*_*s*_	10–400 (ng/mL.cell.day)	[14]
	PDGF consumption rate	*r*_*c*_	(0–1)r_s_ (ng/mL.cell.day)	-
PDGF response	Autocrine boost	*p*_*a*_	0.1–50 (ng/mL)	[14]
	Half max proliferation response	*K*_*p*_	5–300 (ng/mL)	[14,23,25]
	Half max migration response	*K*_*m*_	5–300 (ng/mL)	[14,23,25]
	Recruited proliferation sensitivity	*β*_*p*_	0.1–1.0	-
	Recruited migration sensitivity	*β*_*m*_	0.1–1.0	-
proliferation	Intermitotic time (*p*_*pot*_^*-1*^*)*	*τ*	20–100 (h)	[14,23,25]
	Std dev intermitotic time	*σ*_*τ*_	0–100 (h)	variable
migration	Migration speed (*m*_*pot*_*)*	*v*	0–100 (μm/h)	[14,25]
	Std dev migration speed	*σ*_*v*_	0–100 (μm/h)	variable

**Fig 4:**
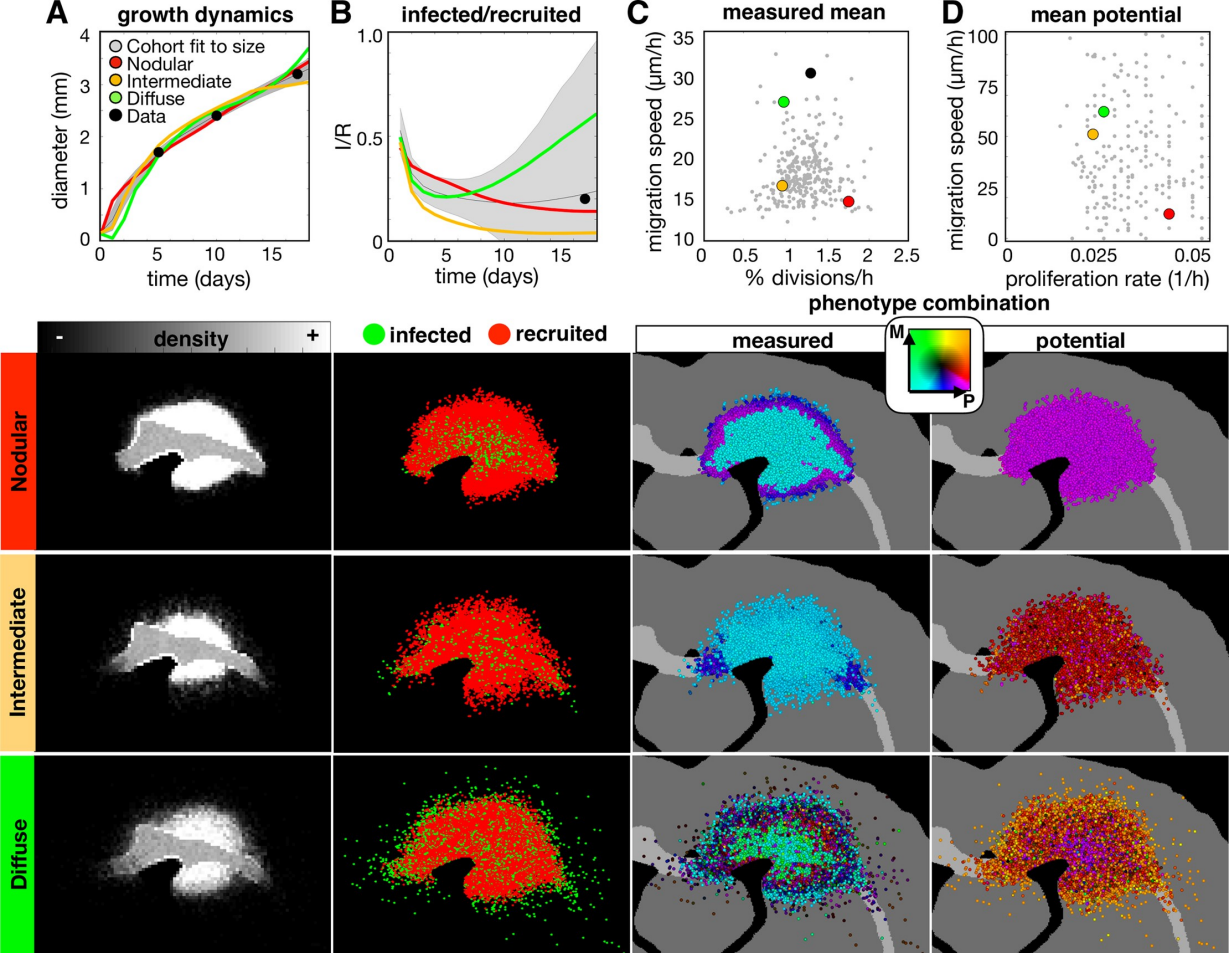
A wide range of *in-silico* tumors fit to the size dynamics from the experimental data. The top row shows the wider variation of the whole cohort of fits, while the spatial distributions below show representative nodular, diffuse, and intermediate density tumors at the 17d time point. The columns correspond to the (A) growth dynamics, (B) ratio of infected to recruited cells over time, (C) measured proliferation rate and migration speed averaged over all cells, and the (D) potential proliferation rate and migration speed (corresponds to the maximum values allowed given a saturated PDGF environment). For each metric, the data points are shown in black, the best fits to the size dynamics of the data are shown in gray (as a mean and standard deviation for dynamic values), and each example tumor is represented in the plots in color (as a mean over 10 runs). Parameter values for each tumor are given in S2 Table. Phenotype are colored according to their combination of proliferation (P) and migration (M) rates according to the color key. Movies are available at jillagal.github.io/multiscaleGBM.

The resulting tumors that fit the size dynamics encompass a broad range of distributions, shapes, and compositions. The results are shown in Fig 4, with plots for metrics going from size dynamics to more smaller scale individual cell metrics (Fig 4A–4D). The diversity of best fits to the growth dynamics is plotted along with 3 examples that represent tumor densities that are more nodular (high density with a very distinct, steep border), diffuse (the tumor core is dense but drops off slowly in density), and intermediate. Spatial distributions for these 3 examples are shown at 17d. The size dynamics in Fig 4A demonstrate that the best fits all have similar trajectories with little overall variation. However, the sizes in the simulation are determined by the average maximum diameter exceeding 10% of the carrying capacity. The many ways that the cells can be distributed and still meet the intended size to match the data are shown below Fig 4A. The nodular tumor is relatively dense with a sharp drop at the edge, whilst the diffuse and intermediate tumors have more fuzzy borders due to a larger portion of cells distributed sparsely throughout the brain. These density differences can be quantified by defining respective tumor core diameters (d_c_ at least 50% cell density) and rim sizes (d_r_ distance between tumor core and at least 1% cell density). On average, the core diameters were 2.3mm, 1.9mm, and 1.9mm for the nodular, intermediate, and diffuse tumors, and the rim sizes were 0.6mm, 1.0mm, and 2.1mm respectively (S3A and S3B Fig).

While the size dynamics were similar amongst these tumors, smaller scale metrics differed substantially. Fig 4B shows the variation in infected (I) and recruited (R) cell numbers. The nodular, intermediate, and diffuse tumors end up with I/R values of 0.17, 0.04, and 0.55, respectively. While both the nodular and intermediate tumors had more recruited cells along the periphery, the intermediate tumor had infected cells that extended farther along the white matter tracts. For the diffuse tumor, infected cells had advanced deep into the brain tissue in all directions.

The combination of average measured trait values covered a large range of values (Fig 4C). The nodular tumor was more proliferative and less migratory, the diffuse tumor was more migratory and less proliferative, and the intermediate tumor had low values for both proliferation and migration. However, these are averages. There are differences in the distribution of individual cells within each of these tumors, which is shown in S3C Fig. There are also differences in the phenotypes along the tumor radius. High cell density, usually in the tumor core, creates a quiescent phenotype (characterized by suspended proliferation), which also varies amongst the tumors. Average values in the measured phenotypes over the tumor radius are shown in S3D Fig.

The potential phenotypes cannot be measured from the data but are of interest as they highlight difference between the realized (measured) and the possible (potential). The potential phenotypes are inherited over generations for each individual cell and represent maximal possible trait values. The nodular tumor is highly proliferative and minimally migratory throughout spatially and temporally. In contrast, the intermediate and migratory tumors are both initialized with similar potential phenotypes on average, however, they present as noticeably distinct tumors due to differences in heterogeneity. These individual cell distributions are shown in S3C and S3D Fig as a heatmap and as an average value along the tumor radius. The effects of selection can be observed in the diffuse tumor, as the highly migratory and proliferative cells are found at the edge of the tumor and the less migratory cells are found in the tumor core.

### Anti-proliferative treatment causes a range of responses *in silico* tumors

**Fig 5:**
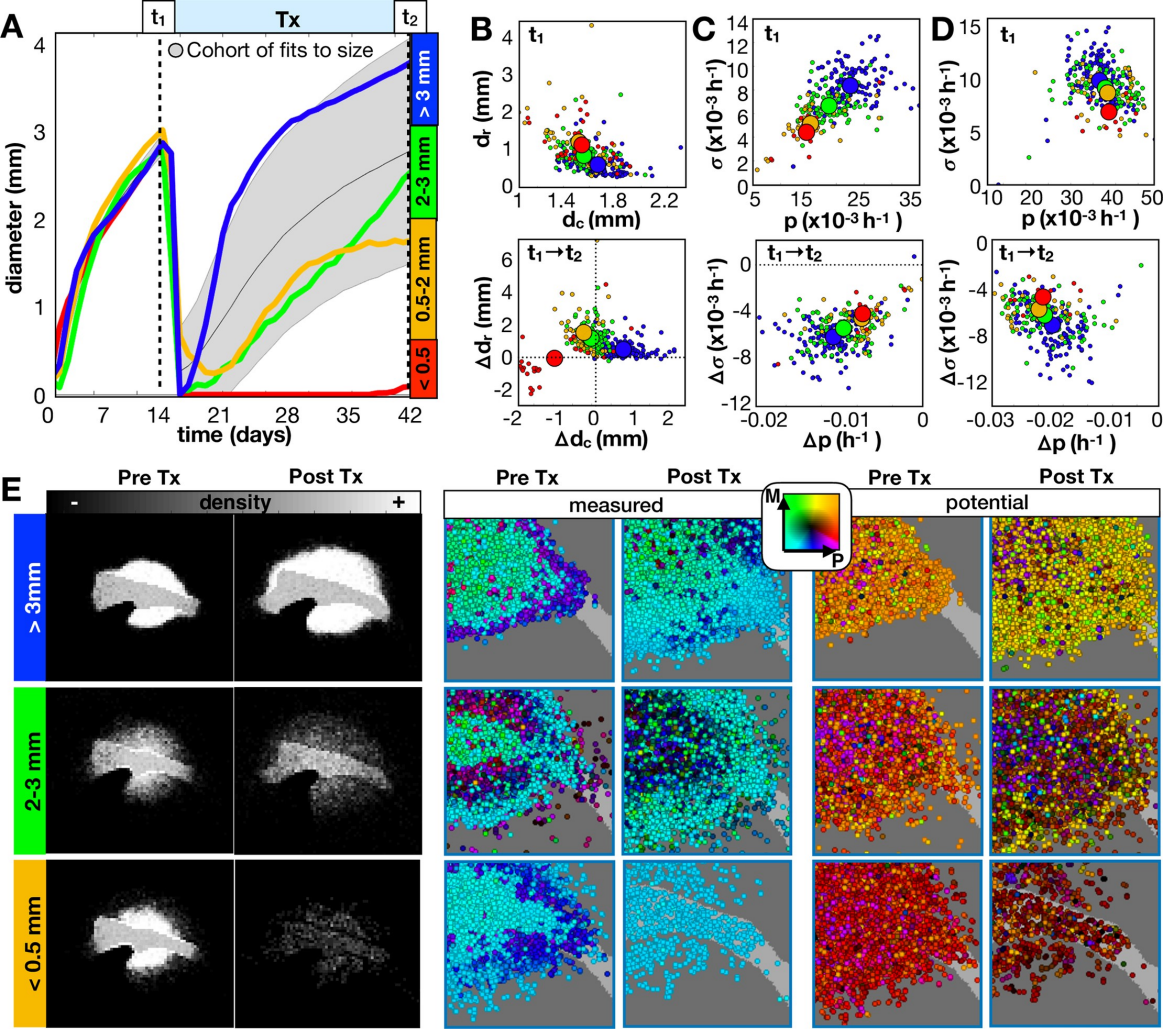
Long term responses of in-silico tumors to an anti-proliferative drug. The drug was applied continuously at 14d until 42d. A) From the growth dynamics, tumors are categorized into 4 outcomes given the final diameter at the end of treatment. We compare the same top 300 fits from Fig 4 and 4 example tumors (including the same 3 tumors from Fig 4) averaged over 10 runs. B-C) Imaging metrics and phenotypes for different outcomes. B) Top: Tumor rim size (d_r_ distance from tumor core to 1% cellular density) vs. tumor core diameter (d_c_ average diameter of at least 50% density) prior to treatment. Bottom: The change in d_r_ vs. the change in d_c_ before and after treatment. C) Top: Standard deviation in measured proliferation rate (*σ*) vs. average measured proliferation rate (p) prior to treatment. Bottom: The change in *σ* vs. the change in p before and after treatment. D) Top: Potential *σ* vs. potential p prior to treatment. Bottom: Change in potential *σ* vs. change in potential p before and after treatment. E) The spatial distributions for the recurrent tumors before and after treatment shown as densities and measured/potential phenotype combinations. Phenotypes are colored according to their combination of proliferation (P) and migration (M) rates according to the color key. Movies are available at jillagal.github.io/multiscaleGBM.

We examined the effect of applying an anti-proliferative drug treatment, which represents a cytotoxic chemotherapy assumed to kill fast proliferating cells. We used a threshold cutoff of 60 hours, and all cells that are not currently quiescent with shorter intermitotic times than the threshold are killed. The drug was applied instantaneously at day 14 and remained on continuously until the simulation was stopped 28 days later. Fig 5 shows the results.

Amongst all tumors in the cohort fit to the same size dynamics, there was a broad range of responses to the anti-proliferative treatment (Fig 5A). In order to compare changes in features over scales, we categorized tumors based on their size at the end of treatment. We can further characterize the tumor imaging profile based on d_c_ and d_r_. From the greater cohort that was fit to the size dynamics, we found that the average nodular tumor (larger d_c_ and smaller d_r_) prior to treatment had a poor outcome (Fig 5B, top), while the more diffuse tumors (smaller d_c_ and larger d_r_) tended to be smaller following treatment. However, there is a lot of noise in this trend, and we even find that the nodular tumor (from Fig 4 and shown in red) had a complete response. The changes in d_c_ and d_r_ for the cohort following treatment are shown in Fig 5B(bottom), and for each recurrent tumor in S4A and S4B Fig.

The measured phenotypes in the cohort showed a clearer separation due to outcome prior to treatment (Fig 5C, top). The worst outcomes had higher measured mean proliferation rates and greater heterogeneity within the tumor. Following treatment, all tumors had slower mean proliferation rates and most showed a reduction in heterogeneity, while the worst outcomes showed the greatest changes in both values (Fig 5C, bottom). The separation between the potential phenotypes due to the final outcome was less clear, however, there was a slight trend toward more heterogeneity within the worst responders prior to treatment (Fig 5D, top). Following treatment, the change in mean potential phenotype was always toward a reduced proliferative capability with the worst outcomes having a greater reduction in proliferative heterogeneity (Fig 5D, bottom). Phenotypic distributions of individual cells within each recurrent tumor are shown in S4C Fig before and after treatment.

The spatial layouts of the recurrent tumors are shown in Fig 5E. All tumors showed marked differences in density profiles and phenotypes following treatment. The rather nodular tumor (top), which represents the worst outcome example, sits in contrast to the best responding tumor Fig 5A that also has a nodular cellular density (seen in Fig 4). This contrasting pair reiterates that tumors with similar imaging profiles can have different underlying phenotypes that greatly affect their response to treatment.

### Cell autonomous heterogeneity causes little difference in tumor growth dynamics but can lead to big differences in response to treatment

**Fig 6:**
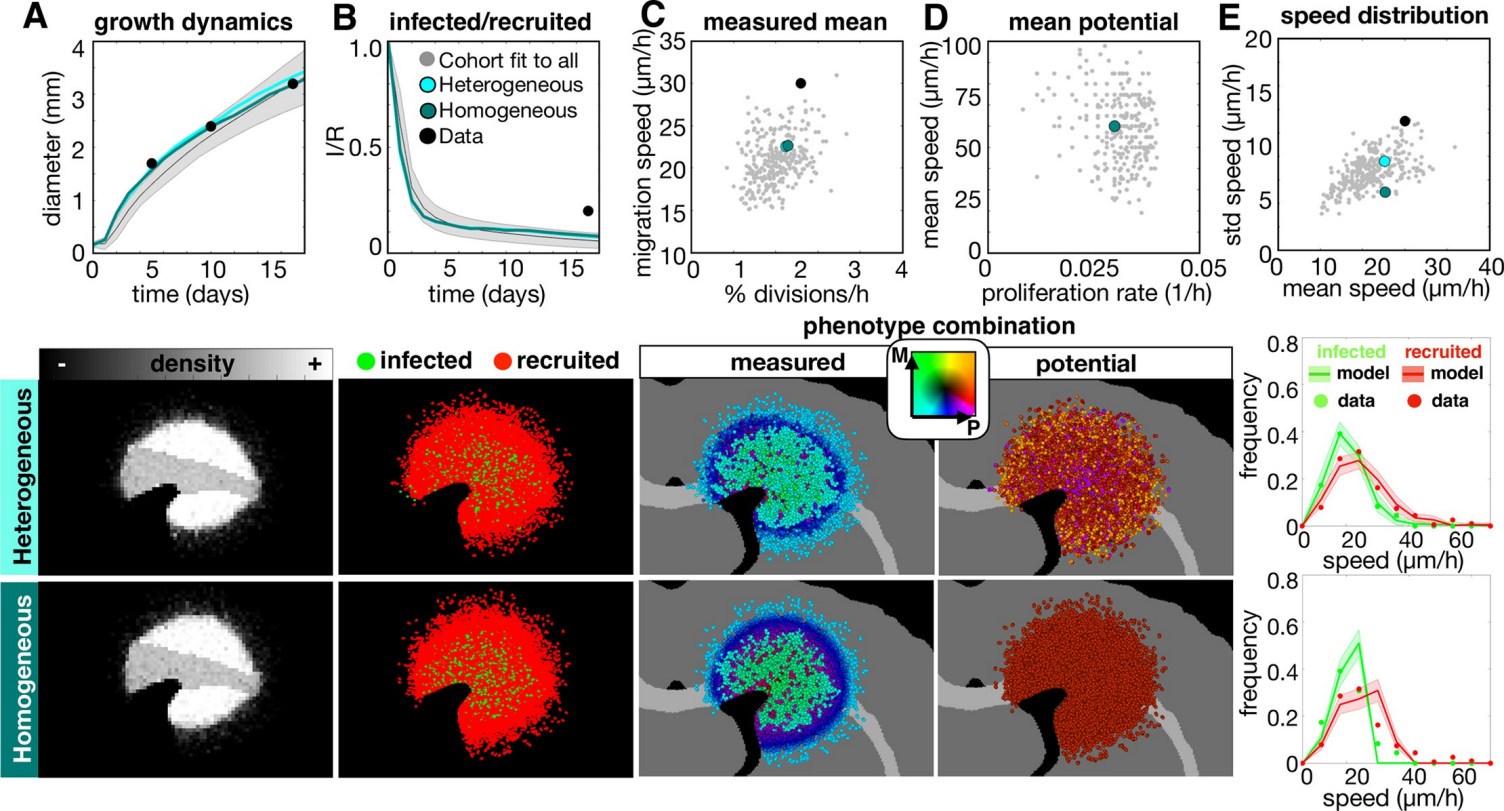
The top fit *in-silico* tumor to the multiscale experimental data using all 16 metrics. The top 300 fits to all data (gray) are compared to the best heterogeneous fit and its homogeneous counterpart (with no variation in potential phenotypes, i.e. *σ*_τ_ = 0, *σ*_v_ = 0). For each metric, the corresponding spatial maps at 17d are shown below. Measured metrics include A) growth dynamics, B) infected/recruited cells over time, C) mean measured proliferation rate and migration speeds at 10d, the D) mean initial potential proliferation rate and migration speed at 10d, and the E) individual cell speed distributions in terms of mean and standard deviation at 10d. The final graphs in column E compare the 10d distributions of speeds of individual tracked cells to the data. Movies are available at jillagal.github.io/multiscaleGBM.

To fit the model at the cell scale, we used the same parameter estimation method that was used to fit the size dynamics with all 16 measured observations from the experimental data. While the final best parameter set didn’t fit all metrics from the *in silico* model equally well to the data, the total weighted error was within 25% for a cohort of parameter sets (S2C and S2D Fig). Given the best fit parameter set from this group, we examined the effect of heterogeneity in the potential phenotype, such that eliminating heterogeneity would cause all observed heterogeneity to be environmentally driven, such as quiescence caused by high cell density and modulation of phenotype by local PDGF concentration. We compared the best fit parameter set (heterogeneous) to one with the same mean potential values for proliferation and migration, but without heterogeneity in these rates, *σ*_τ_ = 0 and *σ*_ν_ = 0, amongst the cells (homogenous) along with the cohort of fits to all the data (Fig 6).

Fitting to all data narrowed the ranges to all metrics shown here with the exception of the size dynamics, which broadened slightly. Both the heterogeneous and homogeneous tumors reasonably fit the size dynamics (Fig 6A) and had similar density distributions (S5A Fig). Both tumors and the larger cohort fit to all data underestimated the infected to recruited ratio (Fig 6B). Both tumors had similar values for the measured proliferation and migration rates (Fig 6C and S5B Fig), showing that the observed heterogeneity is largely influenced by environmental drivers such as tumor density and PDGF concentration. Because the PDGF is highly concentrated at the tumor core and drops off at the tumor edge, the measured proliferation and migration rates are high in the tumor core and reduce with the PDGF concentration (S5C Fig), which agrees with the experimental data. Both tumors were initialized with the same mean trait values (Fig 6D), but the spatial distribution of potential trait values shows that heterogeneity in potential phenotypes can be present without manifesting any noticeable differences in the measured phenotypes. We also found differences in the distribution of individual cell speeds. The mean and standard deviation of speeds fit better when heterogeneity is present than when it is not (Fig 6E), and comparing the distributions, which were averaged over 10 runs, further emphasizes this point (column 6E, lower). The *in silico* measurements for the heterogeneous tumor fit the data by not just matching to the peak, but also capturing the long tail of the distribution. The distribution for the homogeneous tumor drops off sharply at high cell speeds, which most likely occurs due to the maximum speed achieved at saturated PDGF levels. Only a small number of highly migratory cells like in the heterogeneous tumor is needed to create the long tail in this distribution.

**Fig 7:**
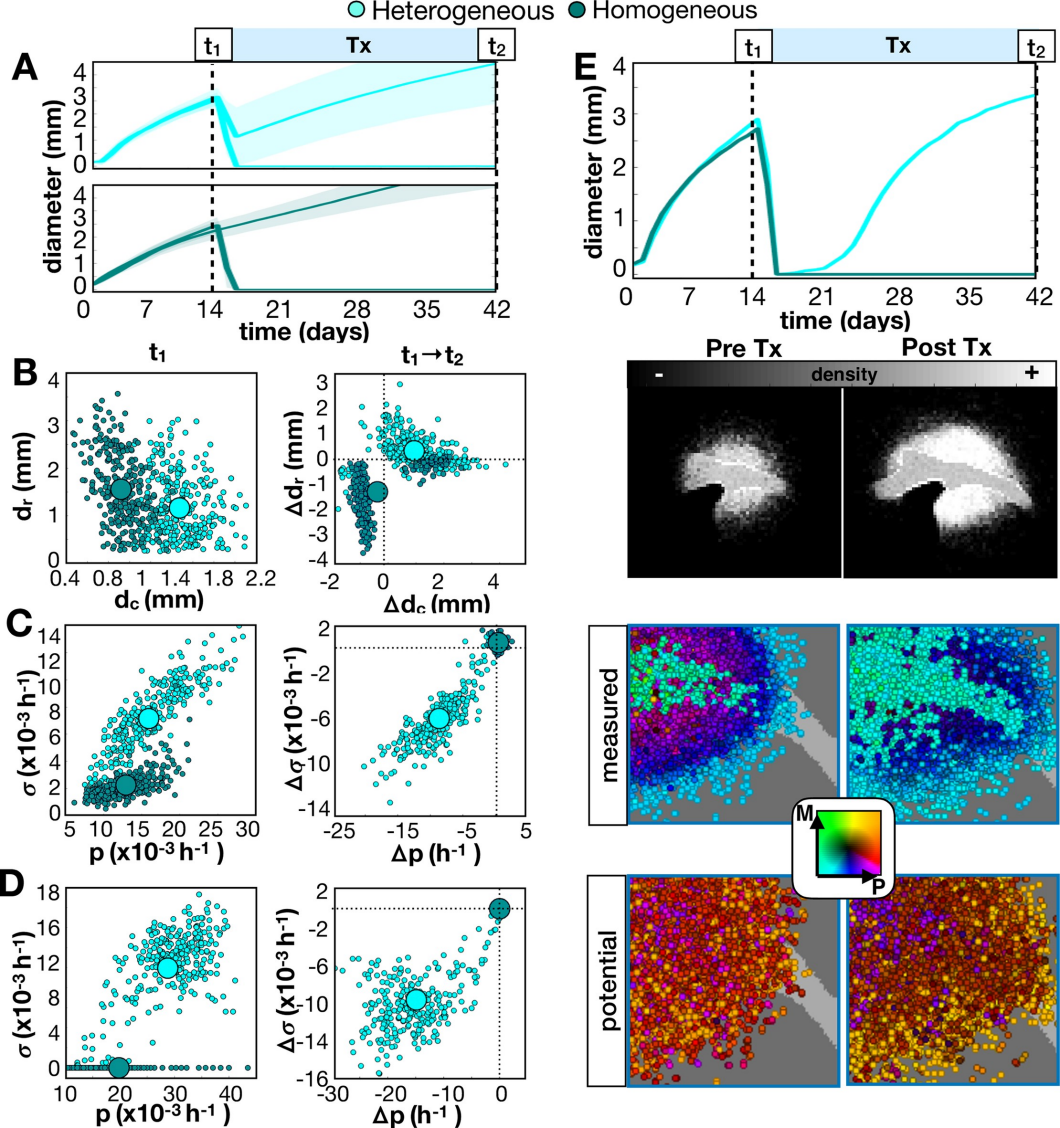
Comparison of long-term responses of heterogeneous and homogeneous in-silico tumors to an anti-proliferative drug. The drug was applied continuously at 14d until 42d. A-D) We compare the cohort fit to all 16 metrics to the same cohort without heterogeneity. A) Growth dynamics. Top: The full cohort is shown as a shaded error plot. Bottom: The best fit from the previous figure is averaged over 10 runs and shown. B) Top: Tumor rim size (d_r_ distance from tumor core to 1% cellular density) vs. tumor core diameter (d_c_ average diameter of 50% density) prior to treatment. Bottom: Change in d_r_ vs. change in d_c_ after treatment. C) Top: Standard deviation in measured proliferation rate (*σ*) vs. average measured proliferation rate (p) prior to treatment. Bottom: Change in *σ* vs. change in p after treatment. D) Top: Standard deviation in potential *σ* vs. average potential p prior to treatment. Bottom: Change in potential *σ* vs. change in potential p after treatment. E) The spatial distribution for the recurrent heterogeneous tumor example before and after treatment shown as densities, measured phenotype combinations and potential phenotype combinations. Phenotypes are colored according to their combination of proliferation (P) and migration (M) rates according to the color key. Movies are available at jillagal.github.io/multiscaleGBM.

If we treat the full cohort and their homogeneous counterparts with an anti-proliferative drug, we find that a heterogeneous tumor generally responds and then recurs (Fig 7A, top), while the homogeneous tumor either responds or does not (Fig 7A, bottom). Further, a sustained complete response was observed in 9% of the heterogeneous tumors versus 76% of the homogenous tumors. From the full cohort, we found that the homogeneous tumors prior to treatment had smaller core diameters (Fig 7B, left) and less heterogeneity in measured and potential proliferation rates (Fig 7C and 7D, left). Most recurrent homogeneous tumors had smaller cores and rims and no phenotypic changes, while most recurrent heterogeneous tumors had larger cores and rims and reduced proliferation, proliferative potential, and heterogeneity (Fig 7B–7D, right). The recurrent tumor example is shown spatially in Fig 7E and quantified in S6A and S6B Fig. The distribution of individual cell phenotypes is shown in S6C Fig.

### Anti-proliferative treatment leads to a less proliferative tumor at recurrence in *in silico* and human tumors

**Fig 8:**
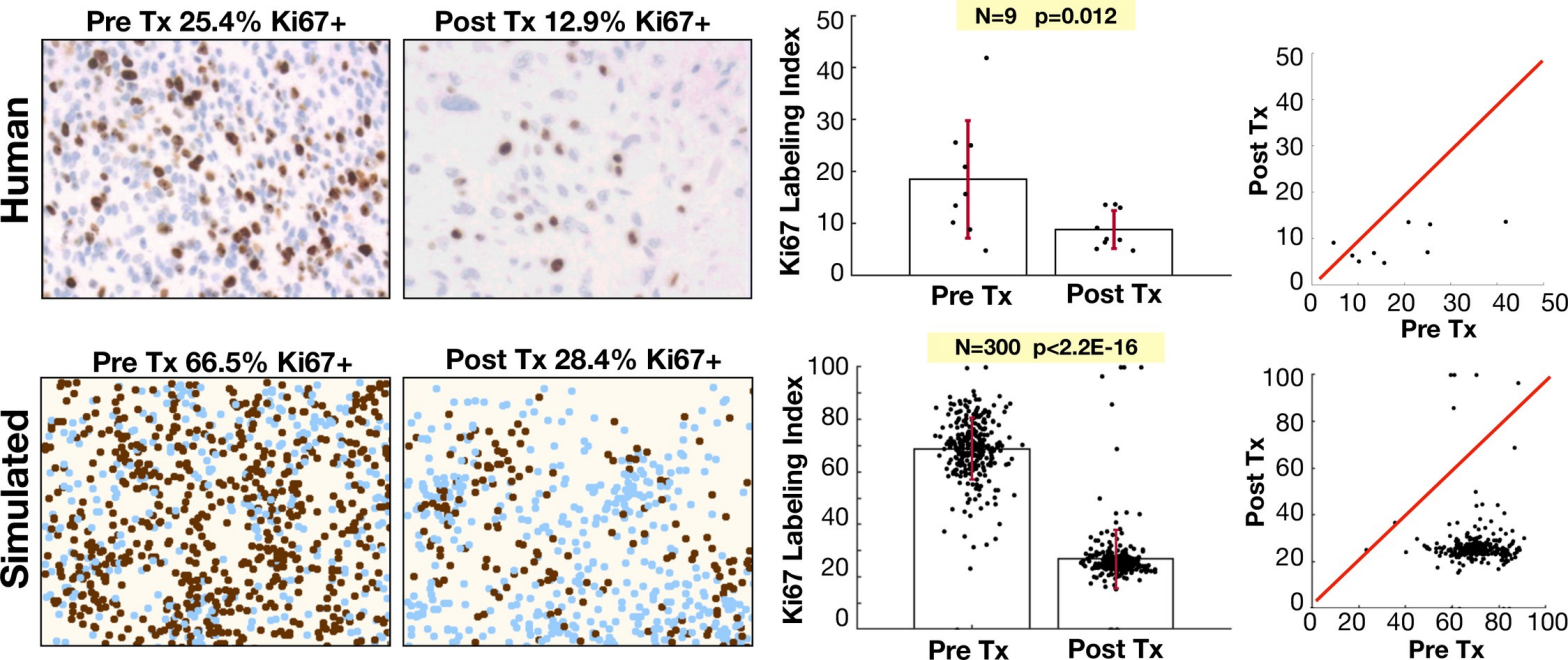
Proliferation is reduced in recurrent tumors. Upper: diagnosis and recurrent tumor specimens from 9 GBM patients stained with Ki-67 antibody indicating proliferating cells. Lower: pre-treatment and post-treatment proliferation index for the virtual cohort fit to size dynamics. Left: Representative pre and post Tx samples. For the patient samples, the labeling index is defined as the % of DAB-stained area out of the total nuclear area for each patient in the region of highest staining density. For the model, we assume that Ki67 is positive only in the last 20 hours of the cell cycle, which is counted as a % in the area of highest activity. Right: Ki67 index is shown with pre and post treatment variation and compared using a Wilcoxon matched-pairs signed rank test. Red line shows the identity line on plot correlating pre and post Tx samples.

Using the mathematical model, we found that antiproliferative drugs caused some degree of tumor recession over all cases tested, but the effect was often only temporary, and the recurring tumor had variable growth dynamics upon recurrence. Furthermore, there was some selection for slightly less proliferative cells, which give rise to recurrence. We also found similar results comparing the proliferating fraction of cells (Ki-67^+^) before and after chemoradiation for nine GBM patients (Fig 8, upper). The proliferating fraction, measured through Ki67 staining, was seen to decrease upon tumor recurrence (p = 0.012, Wilcoxon matched-pairs signed rank test). In these cases, recurrence was defined as the first instance of measurable growth of the lesion on MRI with a clinical determination of disease progression resulting in a change of therapy, excluding pseudo-progression, in which the disease appears to progress and subsequently regress without change in treatment [59,60]. Patients that demonstrated multifocal recurrence defined by multiple lesions not contiguous on MRI were excluded. In the computational model, we also found a reduction following an anti-proliferative treatment in a similar metric for Ki67 for the full cohort fit to the size dynamics (Fig 8, lower). The calculation of Ki67 in the computational model assumes that slower cycling cells spend most of their time in G_0_ (not expressing Ki67). This assumption still resulted in absolute values for Ki67 to be ~2.5 times higher in the computational model, however, the relative changes following treatment are nearly the same as in the human tumors.

### Anti-migratory and anti-proliferative treatment combinations may improve outcomes in some *in silico* tumors

Anti-migratory drugs are an attractive option for very diffuse tumors to try to prevent further invasion into the brain tissue. We examined the effects of an anti-migratory treatment, represented as any agent that slows/stops the migration ability of cells [61,62]. We simulated this treatment by slowing the migration speeds of all cells to 10% of their original speed. We compared an anti-proliferative treatment alone (AP), an anti-migratory treatment alone (AM), and an anti-proliferative and anti-migratory combination (AP+AM). We examined the effect of these treatments on the diffuse tumor from Fig 4 as a prime example for an invasive tumor that could benefit from these treatments.

**Fig 9:**
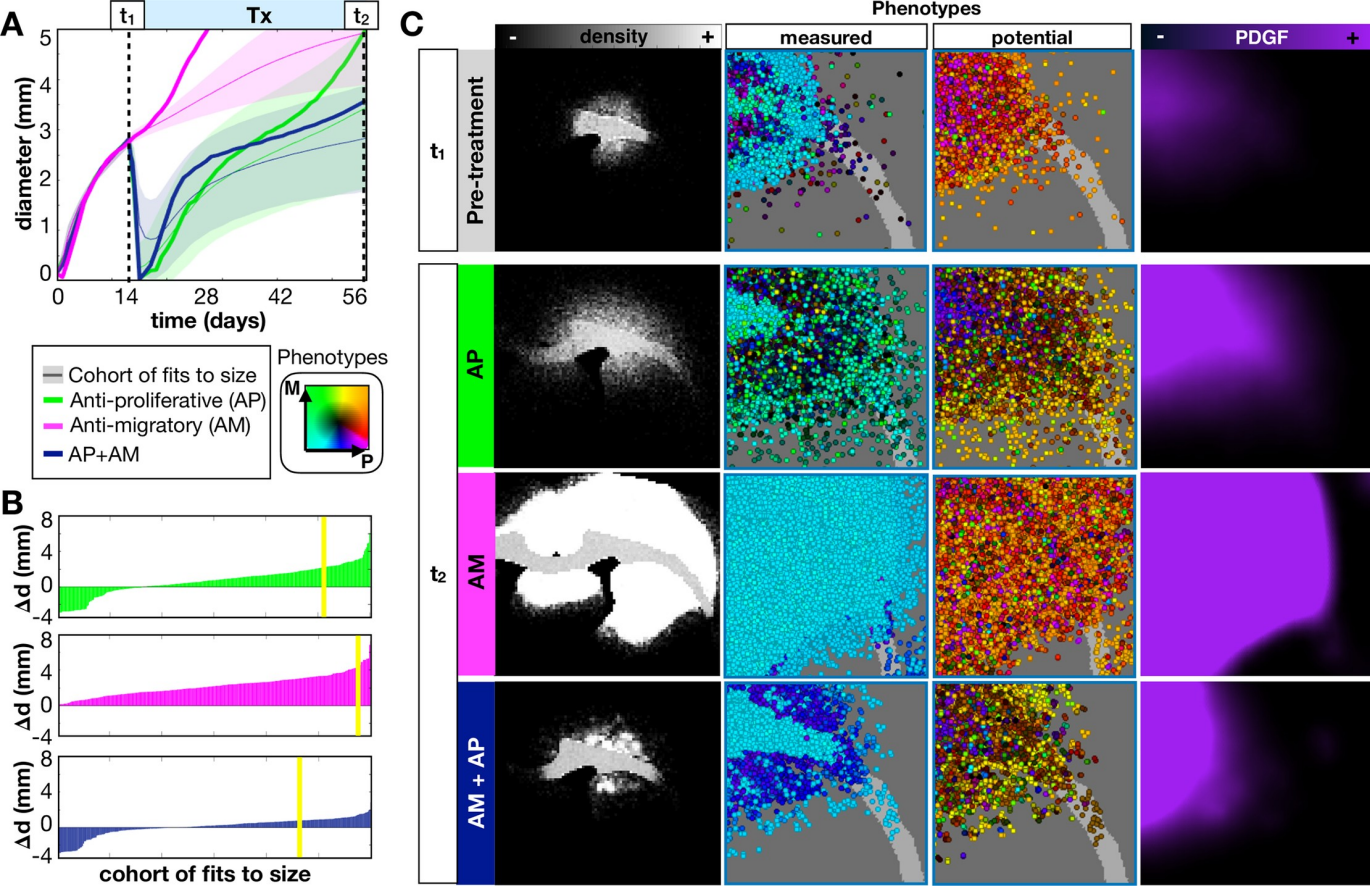
*In-silico* tumors treated with an anti-proliferative drug (AP), anti-migratory drug (AM), or an anti-proliferative, anti-migratory drug combination (AP+AM). The drug is applied continuously at 14d until 28d. A) We show the growth dynamics for the AP, AM, and AP+AM treatments for the top 300 fits to the size dynamics. The average response (from 10 runs) to each treatment of the same diffuse tumor from the previous sections is also shown. B) Waterfall plot of the changes in tumor diameter from t_1_ to t_2_ for the cohort of top 300 fits to size when treated with AP (top) AM (middle), and AP+AM (bottom) treatments. The response of the diffuse tumor to these treatments is shown as a yellow line. C) Treating just the diffuse tumor example, we show representative spatial density distributions, the measured and potential phenotype distributions (colored according to the key), and the PDGF distribution. Movies are available at jillagal.github.io/multiscaleGBM.

The *in silico* results show that the AM treatment alone is not successful in slowing the growth of most tumors, and the diffuse tumor grows especially fast under this treatment (Fig 9A). Compared to the AP treatment, most *in silico* tumors do not do as well on AP+AM treatment at first, but appear to catch up over long applications of treatment.

The full cohort of *in silico* tumors fit to the size dynamics was examined for their response to the different treatments in Fig 9B. We plot the change in tumor diameter before and after each treatment and see that a reduction in diameter is observed with 27% under AP, 0% with AM, and 36% with AP+AM. However, only 8% actually showed a complete response with either AP or AP+AM. Although in most cases, AP+AM resulted in better or similar outcomes than with AP alone (S7 Fig), in some cases, such as the representative diffuse tumor, a better response was seen with AP alone.

The response of diffuse tumor to each treatment is further examined in Fig 9C. Prior to treatment, the tumor had a mean core diameter d_c_ = 1.5mm with a mean rim size d_r_ = 1.4 mm (see also S8A Fig). With the AP treatment alone, the tumor appears to stay smaller for longer after treatment, but this measurement ignores many cells that invaded deep into the brain tissue under the imaging density threshold (at 41d, d_c_ = 2.6mm d_r_ = 2.8mm). With the AP treatment cells continue to migrate into the tissue, and slower proliferating cells are selected for. With the AM treatment, the tumor grows very large since there is no killing taking place, but since the migration has essentially been turned off, growth is driven by proliferation alone rather than proliferation and dispersion (at 41d, d_c_ = 6.3mm d_r_ = 0.5mm). AM treatment selects for cells with high proliferative and migratory potential since they were previously selected for during growth and already populate the outer edges when migration is shut off (see also S8B Fig). The PDGF concentration also becomes saturated in the tissue mediated by lack of cell dispersal, which further drives tumor growth. With the AP+AM treatment, the tumor is observed to be about the same size as the AP treatment (at 41d, d_c_ = 2.3mm d_r_ = 1.3mm), but the tumor was more cohesive and less diffuse. There was selection, again, for less proliferative cells after the AP+AM treatment, and the PDGF concentration was saturated within the tumor core. While the AP+AM treatment worked well over this time period for this tumor, a balance that needs to be made between preventing the widespread distribution of cells into the brain tissue and preventing the buildup of growth factor concentrations to such saturated levels that causes more aggressive cell proliferation at the tumor core.

## Discussion

Tumor heterogeneity is fundamental to treatment success or failure. When predicting a tumor’s long-term response to treatment (observed on serial clinical imaging such as MRI), it is imperative to consider not just the change in the tumor size but also the variation in single cell phenotypes and heterogeneity in the environment. Our results suggest that growth rates alone are not enough to predict drug response; the tumor shape, density, and phenotypic and genotypic compositions can all signify characteristics of the underlying dynamics that affect longer term responses to therapy.

A tumor’s environmental context can play a huge role in malignant progression [5,39]. We found through experiment and simulation that phenotypic heterogeneity is highly modulated by the environmental context. The local environment creates larger scale variations in the observed phenotypes that might be inhibiting, from factors such as lack of space or resources caused by a high cell density, or stimulatory, such as an overabundance of growth factors. These large-scale variations can give insight on environmental niches formed throughout the tumor. At the imaging scale, spatial variations can be quantified to reveal habitats and predict treatment response. Radiomic imaging does just that, because nuances in the shape, morphology, and texture of tumor density maps gives more information than size dynamics alone [3,6–8,18].

### Knowledge of intratumoral heterogeneity is required to predict patterns of treatment response and recurrence

Our results suggest that tumor heterogeneity is also not strictly a factor determined by the microenvironment, but a combination of cell intrinsic drivers and the environmental context. *In silico* tumors that were fit to the same growth dynamics with similar density distributions displayed a huge variation in underlying phenotypes (Fig 4). Furthermore, measurements at the single cell level do not necessarily match up with the potential behavior that cells could achieve given a different environmental context. It is often only after big changes in the tumor microenvironment, such as during therapy, that intrinsic variations at the single cell scale become apparent through natural selection (Fig 5). Importantly, our data suggest that more information on single cell heterogeneity before treatment can lead to better treatment decisions. By fitting the *in silico* model to all of the experimental data, from bulk to single cell metrics, we found a best fit parameter set that resulted in a tumor with heterogeneity in the proliferative and migratory potential (Fig 6). The best fit responded to an anti-proliferative drug but ultimately resulted in recurrence (Fig 7). Eliminating the potential phenotypic heterogeneity in the best fit tumor did not drastically alter the resulting growth dynamics, yet upon exposure to the anti-proliferative treatment there was a complete response. Only at the single cell scale level (Fig 6E) were we able to distinguish these two tumors that ultimately had divergent fates. From this result, it is clear that some degree of single cell observation could aid in the prediction of recurrence and a possible alteration of treatment strategy.

### Model prediction for response to anti-proliferative treatment is recapitulated in human patients

Based on our mathematical modeling results suggesting a diversity of phenotypes in response to treatment, we carefully investigated the role of anti-proliferative treatments since they form the basis of the vast majority of traditional anti-cancer treatments (e.g. radiation and chemotherapies). When fitting the mathematical model to the cell level and tissue level data, we found a consistent pattern of decreased proliferation in simulated recurrent tumors. This finding was recapitulated when we compared a histological marker for proliferation in human GBM patients at diagnosis and recurrence following chemoradiation (Fig 8).

### Model predicts anti-migratory therapy may have limited impact as a monotherapy

Due to the invasiveness of GBM, the use of anti-migratory drugs is appealing [61,63–66]. However, the *in silico* model suggests that anti-migratory drugs do not help when the tumor is largely driven by environmental factors (Fig 9). Moreover, stopping migration also prevented the widespread dispersal of PDGF, leading to more proliferative tumors due to local accumulation of PDGF. This result indicates that, for this type of tumor, anti-migratory therapy alone is not significantly helpful. However, under the right conditions, it might be useful in combination with an anti-proliferative treatment or as a primer for an anti-proliferative drug. The anti-migratory drug was seen to select for more proliferative cells, so perhaps it could be used prior to an anti-proliferative treatment to select for more sensitive cells. Combining these treatments with an anti-PDGF drug could also help, to stop the response to environmental driving force in the first place [67].

### Model design limits interpretation of other biological mechanisms

In our model system we focused on phenotypic heterogeneity within a population of individual cells, which are modulated by the environment through cell density variation, the white/gray matter environment, and PDGF gradients. In order to simplify an already complex model that focuses on the relationship between cell autonomous heterogeneity and environmentally driven heterogeneity due to the growth factor, we excluded some significant drivers of environmental variation such as the angiogenic response, hypoxia, and necrosis [5,68,69]. These are important components in the formation and progression of GBM in particular, however, in order to fit the *in silico* model to the experimental data, we assumed that these factors played a backseat compared to the driving force of PDGF. This was confirmed by the strong sensitivity of parameter for the consumption rate of PDGF, which was quickly pushed to low values by the estimation algorithm. The PDGF-driven rat model grows incredibly fast due to fast proliferation, invasion, and recruitment of a large portion of resident progenitor cells by paracrine growth factor stimulation. While the experimental rat model represents an extreme case compared to human glioblastoma, it is consistent and reproducible, making it a useful tool for controlled data generation to study behavioral heterogeneity. The human disease is generally less aggressive [70], but represents a more heterogeneous group of tumors. The *in silico* model, though calibrated on rat model, sets up an initial framework for addressing heterogeneity in cell traits on multiple scales and within the context of living brain tissue.

### A proliferation-migration dichotomy was not observed in the experimental data

We also made assumptions on the available phenotypes in this model, focusing on the most apparently important traits in GBM: proliferation rate and migration speed. A number of models and experiments find a constraint for an individual cell to be both fast proliferating and fast migrating, the idea of go-or-grow [71,72]. Though it may make sense in the context of limited resources that a cell must divert energy from one task to another, perhaps a tradeoff should not be observed in this model where the environment is rich in growth factors. Furthermore, we found no dichotomy in the experimental data to warrant this assumption, and in fact the opposite was observed. Cells that had divided within the observation period also had a migration speed distribution shifted toward higher speeds. On the other hand, *in silico* tumors with the same size dynamics tended to have measured mean proliferation and migration values that were not often both simultaneously high (Fig 4C), even though individual cells within the population had both fast proliferation and migration rates (S4C Fig). This was tested computationally, by separating the population into growers (fast proliferators that did not move) and goers (migrating cells that proliferate slowly), and we observed a poorer fit than when no such separation existed (S9 Fig). While the migration speed distributions fit well, the constraint on the two populations led to a poor fit for other parameters (S10 Fig). It is possible that the proliferation-migration dichotomy is actually a consequence of environmental variation rather than a cell autonomous feature as seen in the model of Scribner *et al* [38]. We also did not consider the impact of phenotypic evolution [13,42]. The *ex vivo* data showed that the recruited cells, driven at least initially by the environment, proliferate and migrate faster than infected cells, which was found in the fully fit *in silico* model, and that the rates of proliferation and migration of recruited progenitor cells also increase over time. The latter observation could not be reiterated in the *in silico* model with natural selection alone with any of the assumptions we investigated concerning tumor heterogeneity (S11 Fig). Allowing phenotypic drift or transformation in the recruited progenitor population in the computational model, which has been reported to occur in other experimental PDGF-driven glioma models [73], may have helped to fit the data better.

### Model suggests knowledge of intratumoral heterogeneity is required to effectively predict response to treatment

The *in silico* model allowed us to explore spatial dynamics of a tumor as a population and as individual cells to track heterogeneity over time and match to the experimental model. It showed that there likely needs to be both environmental and cell autonomous heterogeneity in order to fit to the smaller scale data, but these components are difficult if not impossible to separate by observation alone in a clinical setting. Specifically, there is no easy way to disentangle the drivers of observed phenotypic behaviors, since intrinsic cell autonomous drivers are modified by cell extrinsic environmental signals that themselves are modified by the cells. Here we have attempted to tackle this question through an integrated approach and hopefully shed light on this complex feedback. Using the hybrid agent-based model, we were able to combine data at different scales to study the environment and phenotypic heterogeneity separately and observe how single cell behavior influenced measurements at different scales. Although the anti-proliferative treatments showed variable responses in the *in silico* model, most were not sustaining and resulted in recurrence with slower proliferating, drug resistant phenotypes. Smarter strategies can be employed when more information is known about the tumor heterogeneity on all scales.

## Supporting information

S1 Methods Single cell analysis. (DOCX)

S2 Methods Hexagonal lattice diffusion. (DOCX)

S3 Methods Matching model to data. (DOCX)

S1 Table Data measured from the rat experiment that was used to fit the model. This contains tumor scale data from imaging, and single cell scale data from the tissue slice data.
(DOCX)

S2 Table Parameter sets used for the example tumors in main text. The parameter ranges are used to search for fits to the data. The nodular, intermediate, and diffuse tumors are found by fitting only to the tumor size data, and the heterogeneous tumor is found by fitting to all of the data. The homogeneous tumor is just the heterogeneous tumor with the variation in proliferation and migration set to zero.
(DOCX)

S1 Fig Behavior of single cells from rat data. A) Wind-Rose plot for infected and progenitor cells at 10d, B) mean squared distance (MSD) for infected and recruited cells at both 2d and 10d, C) distribution of mean migrations speeds, calculated as the total distance travelled over the total time spent moving, at 2d and 10d (mean values, 2d: 24.4μm/h, 10d: 22.6μm/h), D) distribution of instantaneous migration speeds, calculated using method in S1 Methods (mean values over both time points, infected: 12.8μm/h, recruited: 16.6μm/h), and E) distribution of turning angles averaged over infected and recruited cells at 10d.
(TIF)

S2 Fig Parameter estimation by matching to data. Values over iterations of the convergence are shown for A) metrics of top 300 fits fit to size dynamics only, B) parameters from the top 300 fits to size dynamics only, C) metrics of top 300 fits using all data, and D) parameters from the top 300 fits using all data. Each iteration is shown starting at light gray and going to black for the final fit. The red dashed line for the metrics indicates the measured data values, while the blue lines and error bars show the mean and standard deviation over iterations for each parameter.
(TIF)

S3 Fig Tumor profiles over different scales at 17d (corresponding to Fig 4). A) Tumor core and rim are determined from density distributions. For the nodular (NOD), intermediate (INT), and diffuse (DIF) tumors, the core is defined as having a cell density of at least 50% of the carrying capacity, while the rim is defined as having a cell density of at least 1% of the carrying capacity. B) Stacked bar plot of average core diameter and average rim diameter over 10 runs. We define the average rim size as the difference between the average rim diameter and the average core diameter. The average core diameters were 2.3mm, 1.9mm and 1.9mm for the nodular, intermediate, and diffuse tumors, and the average rim sizes were 0.6mm, 1.0mm, and 2.1mm, respectively. C) The measured and potential phenotype combinations for all non-quiescent cells within a single tumor are shown as a scatter plot. The color and location of each dot gives its proliferation rate and migration speed for each cell. The size of the circle is proportional to the number of cells with that phenotype combination, while a white dot marks the mean of the population. D) Spatial phenotype distributions along the radius of the tumor. The average values over 10 runs are plotted for measured proliferation and migration rates and potential proliferation and migration rates.
(TIF)

S4 Fig Changes in tumor profiles following an anti-proliferative treatment (corresponding to Fig 5E). We compare the density distributions and single cell distributions of recurrent tumors of different diameters (>3mm, 2-3mm, and 0.5-2mm). A) The cellular density distributions define the core size (average diameter with a cell density of at least 50% of the carrying capacity) and the rim size (average diameter with a cell density of at least 1% of the carrying capacity). B) Stacked bar plot of average core diameter and rim diameter before and after treatment (over 10 runs). C) The measured and potential phenotype combinations for all non-quiescent cells within a single tumor are shown as a scatter plot. The color and location of each dot gives its proliferation rate and migration speed for each cell. The size of the circle is proportional to the number of cells with that phenotype combination, while a white dot marks the mean of the population.
(TIF)

S5 Fig Tumor profiles over different scales at 17d (corresponding to Fig 6E). A) Tumor core and rim are determined from density distributions. The core is defined as having a cell density of at least 50% of the carrying capacity, while the rim is defined as having a cell density of at least 1% of the carrying capacity. For both tumors, the average core size was 1.9mm and average rim size was 0.6mm. B) The measured and potential phenotype combinations for all non-quiescent cells within a single tumor are shown as a scatter plot. The color and location of each dot gives its proliferation rate and migration speed for each cell. The size of the circle is proportional to the number of cells with that phenotype combination, while a white dot marks the mean of the population. C) Spatial phenotype distributions along the radius of the tumor at 17d. The average values over 10 runs are plotted for measured proliferation and migration rates and potential proliferation and migration rates.
(TIF)

S6 Fig Changes in tumor profiles following an anti-proliferative treatment (from Fig 7E). We compare the density distributions and single cell distributions of the recurrent heterogenous tumor before and after treatment. A) The cellular density distributions define the core size (average diameter with a cell density of at least 50% of the carrying capacity) and the rim size (average diameter with a cell density of at least 1% of the carrying capacity). B) The measured and potential phenotype combinations for all non-quiescent cells within a single tumor are shown as a scatter plot. The color and location of each dot gives its proliferation rate and migration speed for each cell. The size of the circle is proportional to the number of cells with that phenotype combination, while a white dot marks the mean of the population. C) Stacked bar plot of average core diameter and rim diameter before and after treatment (over 10 runs).
(TIF)

S7 Fig Correlation between treatment outcomes over cohort of simulated tumors. We show the distribution of response as A) a waterfall plot with each treatment sorted ranked from best to worst response and B) a waterfall plot for AP treatment sorted ranked from best to worst response but preserving the correlation of how each tumor responds to the other treatments. The yellow line shows the responses for the diffuse tumor from Fig 9. C) Comparison of the responses for AP treatment alone to AP+AM combination treatment. The red line shows where the response is the same for both treatments.
(TIF)

S8 Fig Changes in tumor profiles following different treatments (corresponding to Fig 9C). We compare the density distributions and single cell distributions of the pre-treatment (Pre Tx) and recurrent diffuse tumor after an anti-proliferative treatment (AP), an anti-migratory treatment (AM), and an anti-proliferative and anti-migratory combination (AP+AM). A) The cellular density distributions define the core size (average diameter with a cell density of at least 50% of the carrying capacity) and the rim size (average diameter with a cell density of at least 1% of the carrying capacity). B) The measured and potential phenotype combinations for all non-quiescent cells within a single tumor are shown as a scatter plot. The color and location of each dot gives its proliferation rate and migration speed for each cell. The size of the circle is proportional to the number of cells with that phenotype combination, while a white dot marks the mean of the population.
(TIF)

S9 Fig Parameter estimation assuming go-or-grow by matching to data. Values over iterations of the convergence are shown for A) metrics of top 300 fits using all data, and B) parameters from the top 300 fits using all data. Each iteration is shown starting at light gray and going to black for the final fit. The red dashed line for the metrics indicates the measured data values, while the blue lines and error bars show the mean and standard deviation over iterations for each parameter. For the “grow” population, τ_grow_ was fit while ν_grow_ = 0, and for the “go” population, τ_grow_ = 200h, while ν_grow_ was fit.
(TIF)

S10 Fig Model fit assuming go-or-grow. We show A) the growth dynamics, B) the infected/recruited ratio, C) the mean proliferation rate and mean migration speed combination, D) the potential trait combinations, and E) the mean and standard deviation of the speed distribution. Lower row, columns A-D show the spatial distributions at day 17 from which each metric is measured. The distribution of migration speeds from the single cell tracks is shown in the lower graph in column E).
(TIF)

S11 Fig Comparison of the measured proliferation rates from data and different instances of the computational model. The error bar shows the resulting proliferation rate for the same best fit parameter set over 10 runs for each instance including: i) heterogeneous tumor: allowed heterogeneity in proliferation and migration, ii) homogeneous tumor: only environmental heterogeneity allowed, and iii) go-or-grow tumor: one cell type was fit to proliferation rate and allowed no migration, and one cell type was fit to migration speed with a slow proliferation rate (200h intermitotic time).
(TIF)
