# Supplementary material for: From cells to tissue: How cell scale heterogeneity impacts glioblastoma growth and treatment response
Source: PLoS Comput Biol. 2020 Feb 26;16(2):e1007672. doi: 10.1371/journal.pcbi.1007672 (PMC7062288; doi:10.1371/journal.pcbi.1007672)
Supplement: S1 Methods — (DOCX) [file pcbi.1007672.s001.docx]

**S1 Methods. Single cell analysis**

From the single cell tracks we quantified the migration behavior as seen below. The speed for each cell over the time period was calculated as the total distance travelled over the total time spent moving. Since the cells frequently stopped for long periods of time, we excluded this from the calculation. Due to uncertainty in the cell’s center we defined a stopped cell as moving less than 5 *µ*m over the sampling time. The turning angles and persistence times were calculated by defining run times punctuated by frequent stops. We defined a single run as i) traveling a distance greater than 5 microns during the sampling time, and ii) continuing in the same direction to within 15 degrees of the original trajectory. A single stop time is just the amount of time spent before moving more than 5 *µ*m. The sampling frequency matters when capturing the observed speed and angle distributions [1,2]. Due to the noisy data, which was recorded every 3 minutes, we sampled in 30-minute intervals, starting at different initial 3-minute time point within the 30-minute time interval, so no data was missed. Using these rules, we calculated turning angles, and persistence times.


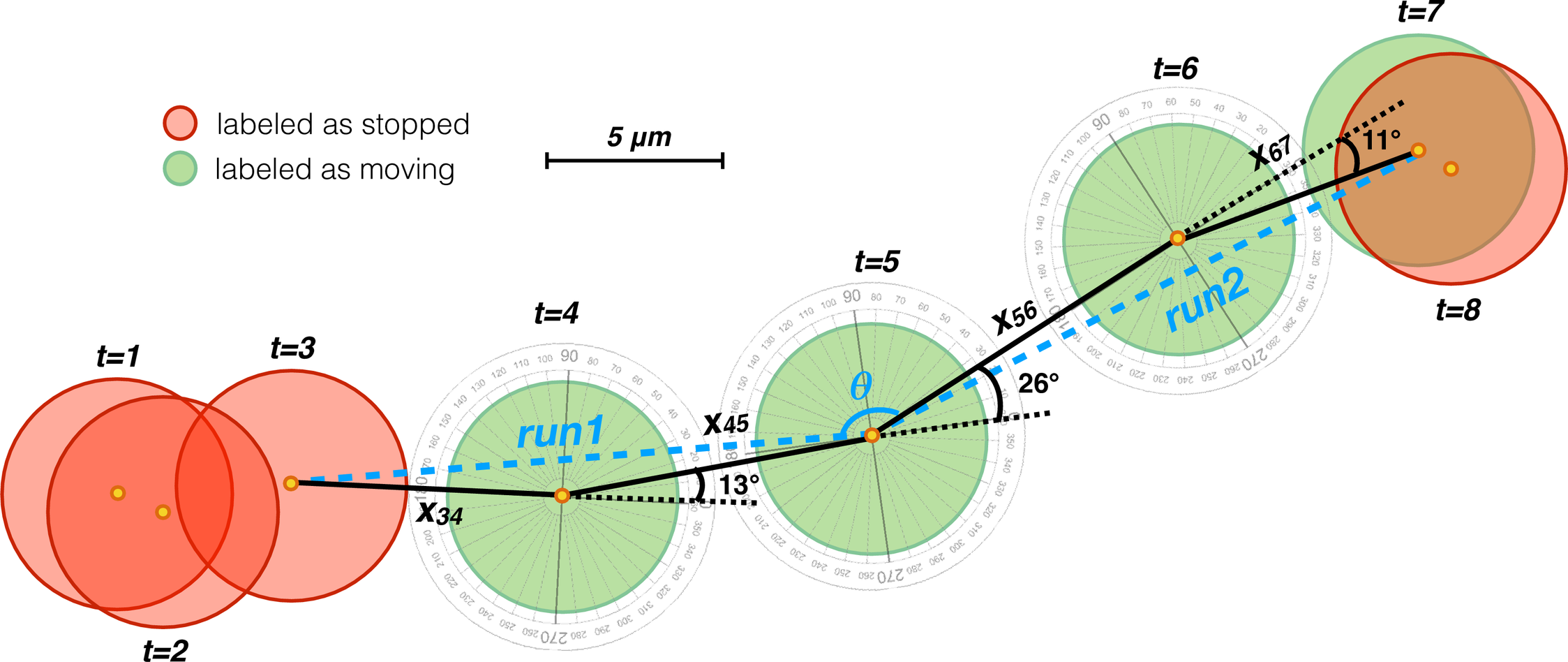


**Cell track data analysis algorithm**. Stops, runs, and turning angles are defined from each cell’s 2D track. The cell speed was calculated from the total distance travelled over the total time travelled (trajectory from solid black lines). If the cell moves greater than 5 𝜇m and does not turn more than 15 degrees during its trajectory, it is considered a single run (two runs and one turning angle labeled in blue).

**References**

1. Rosser G, Fletcher AG, Maini PK, Baker RE. The effect of sampling rate on observed statistics in a correlated random walk. J R Soc Interface. 2013;10(85):24–9.

2. Dickinson RB, Tranquillo RT. Optimal estimation of cell movement indices from the statistical analysis of cell tracking data. AIChE J. 1993;39(12):1995–2010.
