# Supplementary material for: From cells to tissue: How cell scale heterogeneity impacts glioblastoma growth and treatment response
Source: PLoS Comput Biol. 2020 Feb 26;16(2):e1007672. doi: 10.1371/journal.pcbi.1007672 (PMC7062288; doi:10.1371/journal.pcbi.1007672)
Supplement: S2 Methods — (DOCX) [file pcbi.1007672.s002.docx]

**S2 Methods. Hexagonal lattice diffusion**

We create a hexagonal lattice to store information on the type of tissue (gray or white matter) and the concentration of PDGF. Since the lattice has a staggered layout, it is indexed according to the scheme shown below. The concentration of PDGF at any time point is determined by adding to the concentration at the previous time point the sum of the differences between all the neighboring lattice sites times the diffusion coefficient and the time step over the distance travelled from the center of one lattice site to its neighbor. For each lattice point *i*, we write the concentration of PDGF as:

$$p_{x,y}\left( t+\Delta t \right)=p_{x,y}\left( t \right)+\frac{D_{p}\Delta t}{{\Delta x}^{2}}\sum_{n=1}^{6} p_{x,y}\left( t \right)-p_{i}\left( t \right)$$

where is Δ*t* the time step, and Δ*x* is the distance from one lattice point to a neighboring one, which is just twice the apothem, so Δ*x* = *r*√3/2 where *r* is the radius of the hexagon, which in this model is also the same size as the radius of a cell. There is no flux at the boundaries, so there is no contribution from off-grid neighbors.


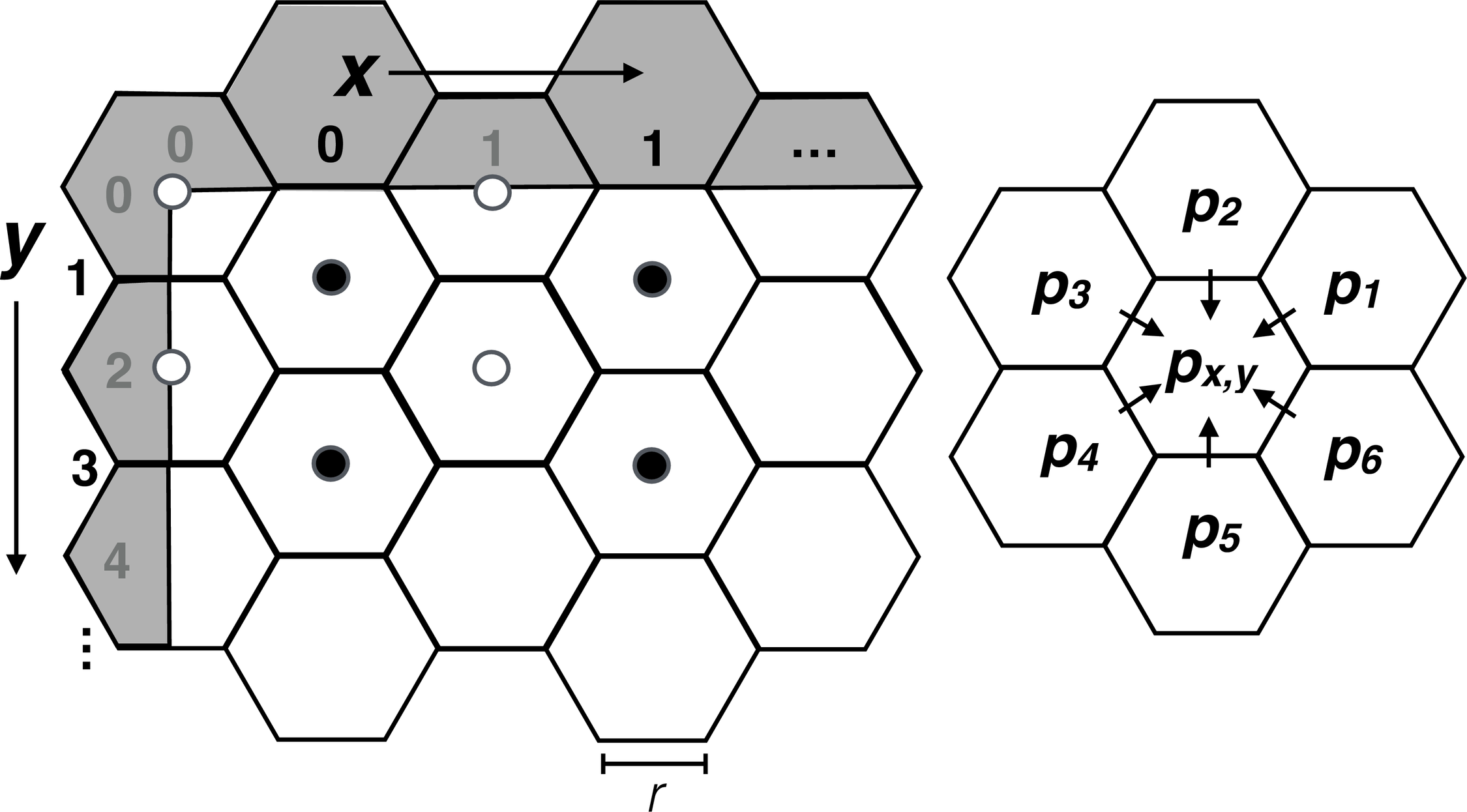


**Hexagonal lattice diffusion.** The lattice points are indexed as shown, with the even columns (black center dots) shifted down halfway in between the even rows (white center dots). Diffusion occurs at each lattice point p_x,y_ between the nearest neighbors only within the boundary of the domain. Off-grid lattice points (gray region) do not contribute to any flux.
