# Supplementary material for: From cells to tissue: How cell scale heterogeneity impacts glioblastoma growth and treatment response
Source: PLoS Comput Biol. 2020 Feb 26;16(2):e1007672. doi: 10.1371/journal.pcbi.1007672 (PMC7062288; doi:10.1371/journal.pcbi.1007672)
Supplement: S3 Methods — (DOCX) [file pcbi.1007672.s003.docx]

**S3 Methods. Matching model to data**

**Model Metrics**

**Tumor size.** Since the tumor consists of cells often spread out with no clear boundary, the tumor size was measured by finding the maximum distance from the center of mass where the cell density is >= 10% of the carrying capacity (averaged over 12 angles).

**I/R*.*** The ratio of infected to recruited cells is calculated, as with the data, by observing the number of each cell type within the tumor core.

**Single cell metrics.** For the single cell metrics, a randomly chosen subset of up to 200 cells were tracked of the initially injected and labeled subset (as opposed to native inactive progenitors) outside of the densest regions (less than 25% of the carrying capacity). These criteria bias the tracks to the tumor edge where it is not too dense to agree with the experimental tracking limitations. Proliferation events and positions of the cells were tracked every 3 min. The measured proliferation rate and migration speeds were calculated by recording the percent of divisions over the observation period and the total distance traveled over the time spent moving, respectively. These values were averaged for infected and recruited cells.

**Convergence scheme**

There are 16 free parameters that are either a) not measured, b) not well determined by experimental estimates, or c) variables in the simulation. These are listed in Table 1 of the main text. To converge on reasonable parameter estimates that result in good fits to the data in S1 Table, we start by drawing 5000 sets of random values for each parameter within the determined ranges. From each parameter set we run the simulation, calculate the output values for each metric, and compare to the data output to get a total error, weighting the size values double, the proliferation rates half, and all else single. We then sort these from least to most error and take the top 10%, excluding any set of parameters that have more than 50% of the total error in one metric, and transfer these directly to the next iteration. For the next 40% of the next iteration, we tweak all of the parameters in the top 10% by a random value sampled from a normal distribution with a standard deviation of 10% of the parameter range. For the final 50% of the next parameter set, we draw randomly from the parameter distribution of the top 10% to introduce new combinations of values. We iterate this procedure until the output error is within 10% for fitting the size dynamics, which turns out to be after 5 iterations, and within 25% for fitting all metrics, which is after 13 iterations.
