# Supplementary material for: From cells to tissue: How cell scale heterogeneity impacts glioblastoma growth and treatment response
Source: PLoS Comput Biol. 2020 Feb 26;16(2):e1007672. doi: 10.1371/journal.pcbi.1007672 (PMC7062288; doi:10.1371/journal.pcbi.1007672)
Supplement: S1 Table — This contains tumor scale data from imaging, and single cell scale data from the tissue slice data. (DOCX) [file pcbi.1007672.s004.docx]

| **PARAMETER** | **SCALE** | **TIME POINT (d)** | **VALUE** | **SOURCES** |
| --- | --- | --- | --- | --- |
| **Diameter (mm)** | tumor | 5 | 1.7 | [1,2] |
|  | tumor | 10 | 2.4 | [1,2] |
|  | tumor | 17 | 3.2 | [1,2] |
| **Ratio I/R** | tumor | 17 | 0.2 | [1] |
| **mean proliferation rate**  **(% cells/h)** | infected cells | 2 | 0.33 | calculated |
|  | recruited cells | 2 | 0.85 | calculated |
|  | infected cells | 10 | 0.83 | calculated |
|  | recruited cells | 10 | 1.89 | calculated |
| **mean migration rate**  **(𝜇m/h)** | infected cells | 2 | 21.3 | calculated |
|  | recruited cells | 2 | 24.9 | calculated |
|  | infected cells | 10 | 20.6 | calculated |
|  | recruited cells | 10 | 25.2 | calculated |
| **standard deviation migration (𝜇m/h)** | infected cells | 2 | 5.7 | calculated |
|  | recruited cells | 2 | 7.6 | calculated |
|  | infected cells | 10 | 5.7 | calculated |
|  | recruited cells | 10 | 8.8 | calculated |

**References**

1. Assanah MC, Lochhead R, Ogden A, Bruce J, Goldman J, Canoll P. Glial Progenitors in Adult White Matter Are Driven to Form Malignant Gliomas by Platelet-Derived Growth Factor-Expressing Retroviruses. J Neurosci. 2006;26(25):6781–90.

2. Massey SC, Assanah MC, Lopez KA, Canoll P, Swanson KR. Glial progenitor cell recruitment drives aggressive glioma growth: mathematical and experimental modelling. J R Soc Interface. 2012;9(73):1757–66.
