# Supplementary material for: From cells to tissue: How cell scale heterogeneity impacts glioblastoma growth and treatment response
Source: PLoS Comput Biol. 2020 Feb 26;16(2):e1007672. doi: 10.1371/journal.pcbi.1007672 (PMC7062288; doi:10.1371/journal.pcbi.1007672)
Supplement: S2 Table — The parameter ranges are used to search for fits to the data. The nodular, intermediate, and diffuse tumors are found by fitting only to the tumor size data, and the heterogeneous tumor is found by fitting to all of the data. The homogeneous tumor is just the heterogeneous tumor with the variation in proliferation and migration set to zero. (DOCX) [file pcbi.1007672.s005.docx]

| **SYMBOL (UNITS)** | **RANGE** | **Nodular** | **Intermediate** | **Diffuse** | **Hetero** | **Homo** |
| --- | --- | --- | --- | --- | --- | --- |
| ***𝜌_R_* (%)** | 0.1-5 | 3.3 | 3.2 | 4.8 | 3.8 | 3.8 |
| ***𝜎_𝜃_* (degrees)** | 0-45 | 23 | 15 | 29 | 41 | 41 |
| ***p_0_* (ng/mL)** | 100-600 | 425 | 425 | 499 | 400 | 400 |
| ***D_p_* (x10^-6^ cm^2^/day)** | 1-1000 | 67 | 165 | 187 | 301 | 301 |
| ***r_d_* (ng/mL.day)** | 0-0.500 | 0.343 | 0.299 | 0.064 | 0.025 | 0.025 |
| ***r_s_* (ng/mL.cell.day)** | 10-400 | 169 | 190 | 115 | 361 | 361 |
| ***r_c_* (% of r_s_)** | 0-100 | 2 | 5 | 20 | 5 | 5 |
| ***p_a_* (ng/mL)** | 0.1-50 | 36.7 | 47.9 | 37.5 | 7.6 | 7.6 |
| ***K_p_* (ng/mL)** | 5-300 | 55 | 183 | 56 | 94 | 94 |
| ***K_m_* (ng/mL)** | 5-300 | 25 | 162 | 56 | 256 | 256 |
| ***𝛽_p_*** | 0.1-1.0 | 0.66 | 0.10 | 0.93 | 0.47 | 0.47 |
| ***𝛽_m_*** | 0.1-1.0 | 0.63 | 0.72 | 0.71 | 0.51 | 0.51 |
| ***𝜏* (h)** | 20-100 | 24 | 45 | 40 | 48 | 48 |
| ***𝜎_𝜏_* (h)** | 0-100 | 3 | 12 | 51 | 5 | 0 |
| ***𝜈* (𝜇m/h)** | 0-100 | 12 | 51 | 62 | 60 | 60 |
| ***𝜎_𝜈_* (𝜇m/h)** | 0-100 | 2 | 9 | 58 | 25 | 0 |
