# Supplementary figures and images for: From cells to tissue: How cell scale heterogeneity impacts glioblastoma growth and treatment response
Source: PLoS Comput Biol. 2020 Feb 26;16(2):e1007672. doi: 10.1371/journal.pcbi.1007672 (PMC7062288; doi:10.1371/journal.pcbi.1007672)

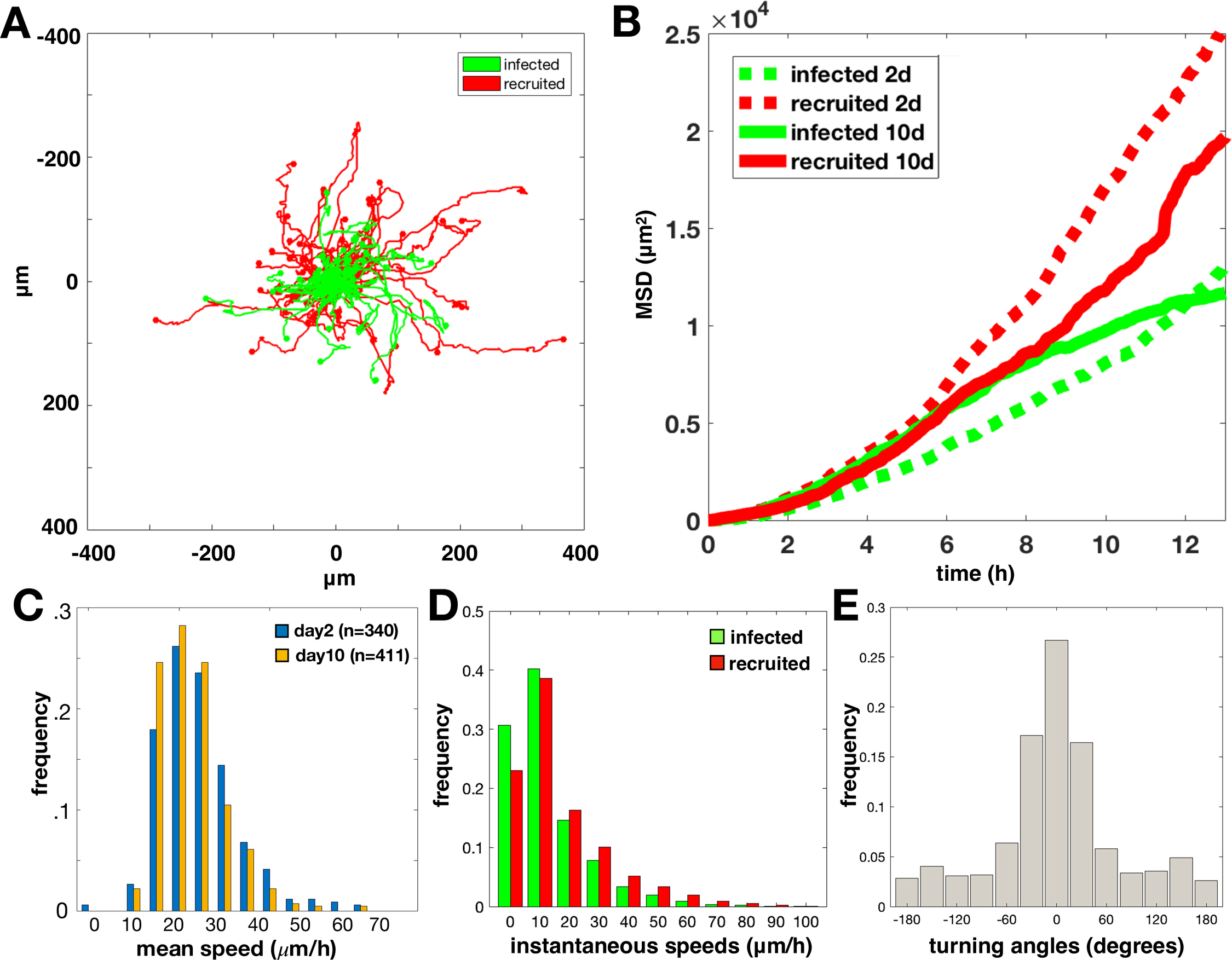

Supplement: S1 Fig — A) Wind-Rose plot for infected and progenitor cells at 10d, B) mean squared distance (MSD) for infected and recruited cells at both 2d and 10d, C) distribution of mean migrations speeds, calculated as the total distance travelled over the total time spent moving, at 2d and 10d (mean values, 2d: 24.4μm/h, 10d: 22.6μm/h), D) distribution of instantaneous migration speeds, calculated using method in S1 Methods (mean values over both time points, infected: 12.8μm/h, recruited: 16.6μm/h), and E) distribution of turning angles averaged over infected and recruited cells at 10d. (TIF) [file pcbi.1007672.s006.tif]

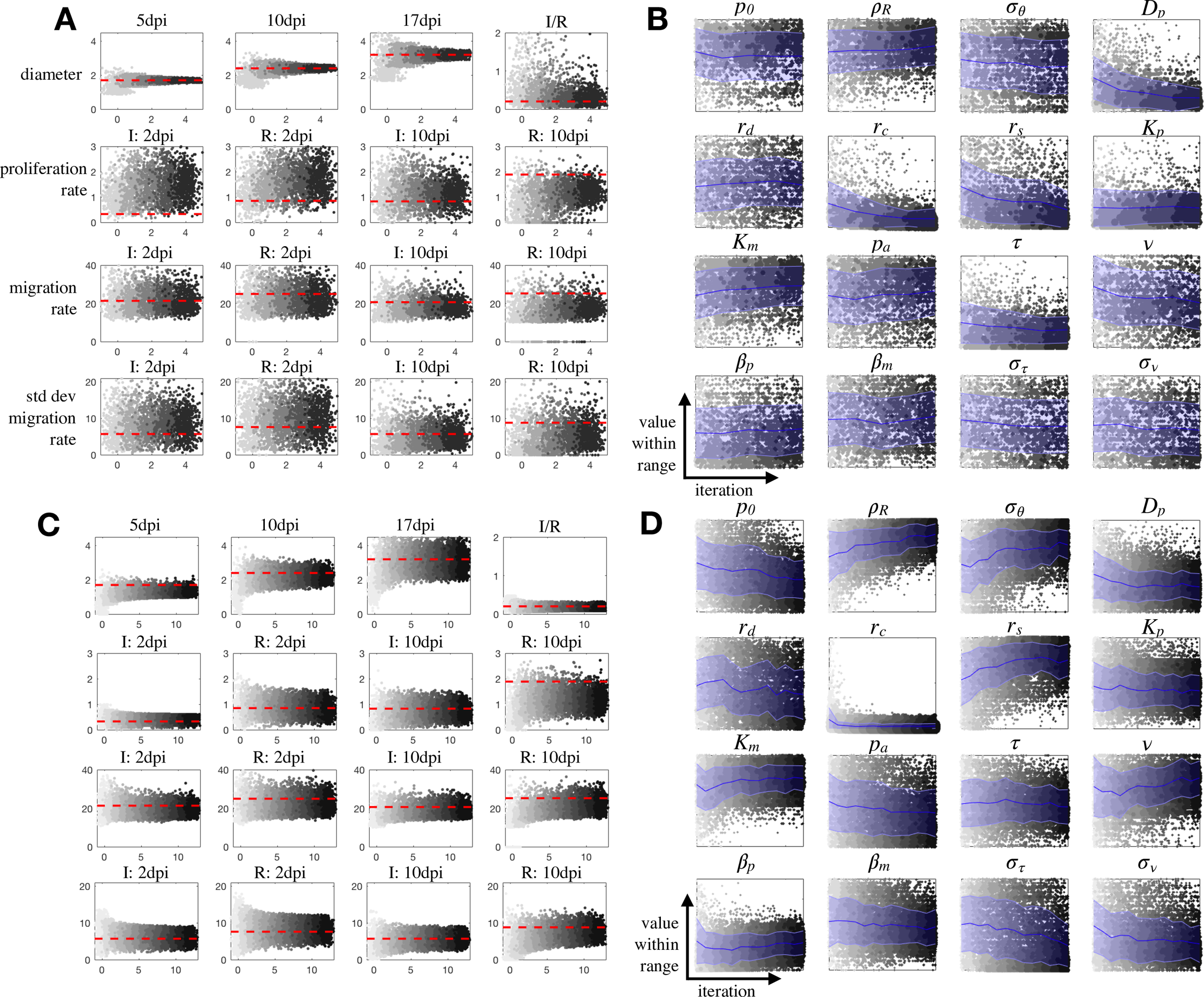

Supplement: S2 Fig — Values over iterations of the convergence are shown for A) metrics of top 300 fits fit to size dynamics only, B) parameters from the top 300 fits to size dynamics only, C) metrics of top 300 fits using all data, and D) parameters from the top 300 fits using all data. Each iteration is shown starting at light gray and going to black for the final fit. The red dashed line for the metrics indicates the measured data values, while the blue lines and error bars show the mean and standard deviation over iterations for each parameter. (TIF) [file pcbi.1007672.s007.tif]

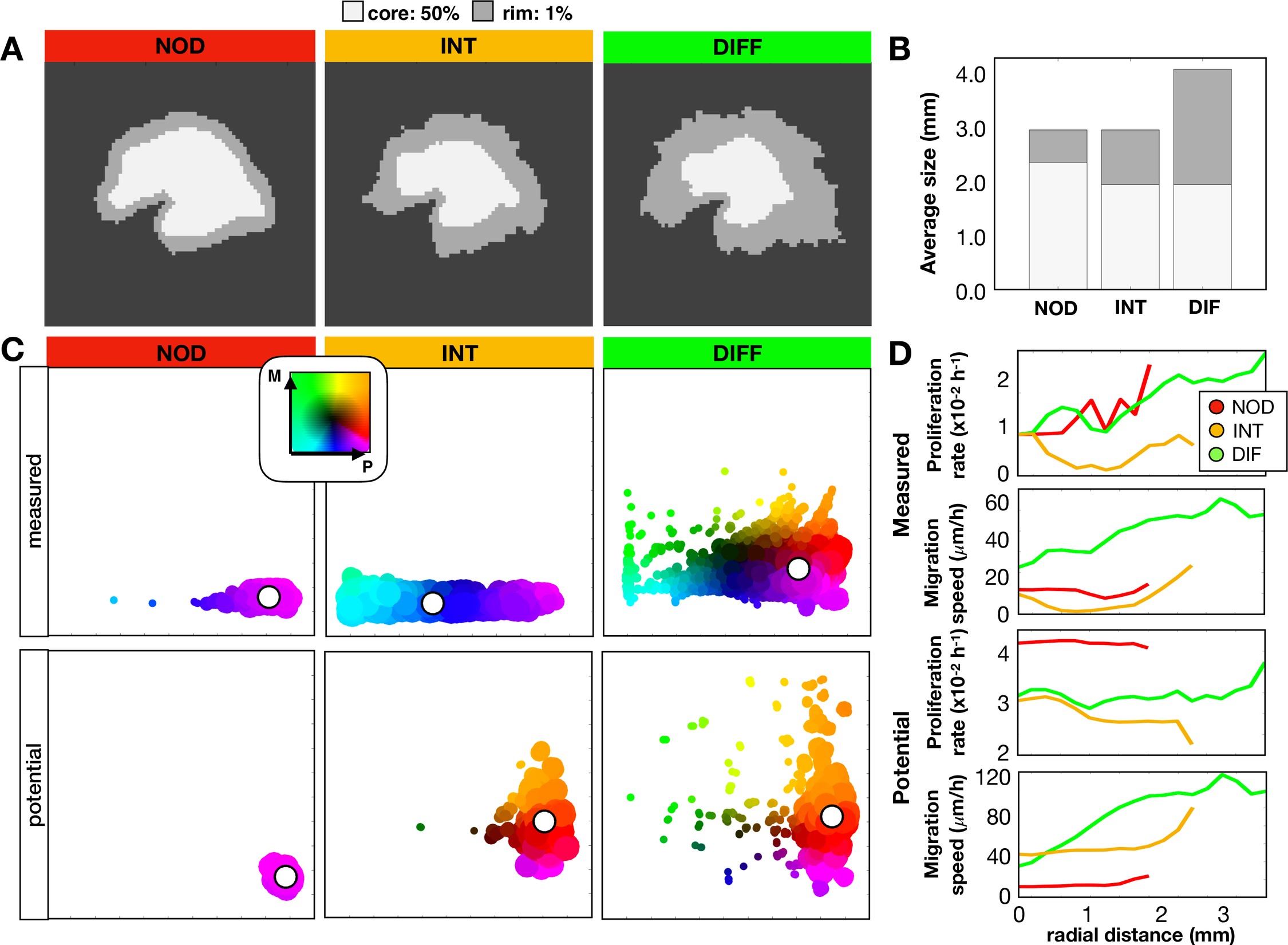

Supplement: S3 Fig — A) Tumor core and rim are determined from density distributions. For the nodular (NOD), intermediate (INT), and diffuse (DIF) tumors, the core is defined as having a cell density of at least 50% of the carrying capacity, while the rim is defined as having a cell density of at least 1% of the carrying capacity. B) Stacked bar plot of average core diameter and average rim diameter over 10 runs. We define the average rim size as the difference between the average rim diameter and the average core diameter. The average core diameters were 2.3mm, 1.9mm and 1.9mm for the nodular, intermediate, and diffuse tumors, and the average rim sizes were 0.6mm, 1.0mm, and 2.1mm, respectively. C) The measured and potential phenotype combinations for all non-quiescent cells within a single tumor are shown as a scatter plot. The color and location of each dot gives its proliferation rate and migration speed for each cell. The size of the circle is proportional to the number of cells with that phenotype combination, while a white dot marks the mean of the population. D) Spatial phenotype distributions along the radius of the tumor. The average values over 10 runs are plotted for measured proliferation and migration rates and potential proliferation and migration rates. (TIF) [file pcbi.1007672.s008.tif]

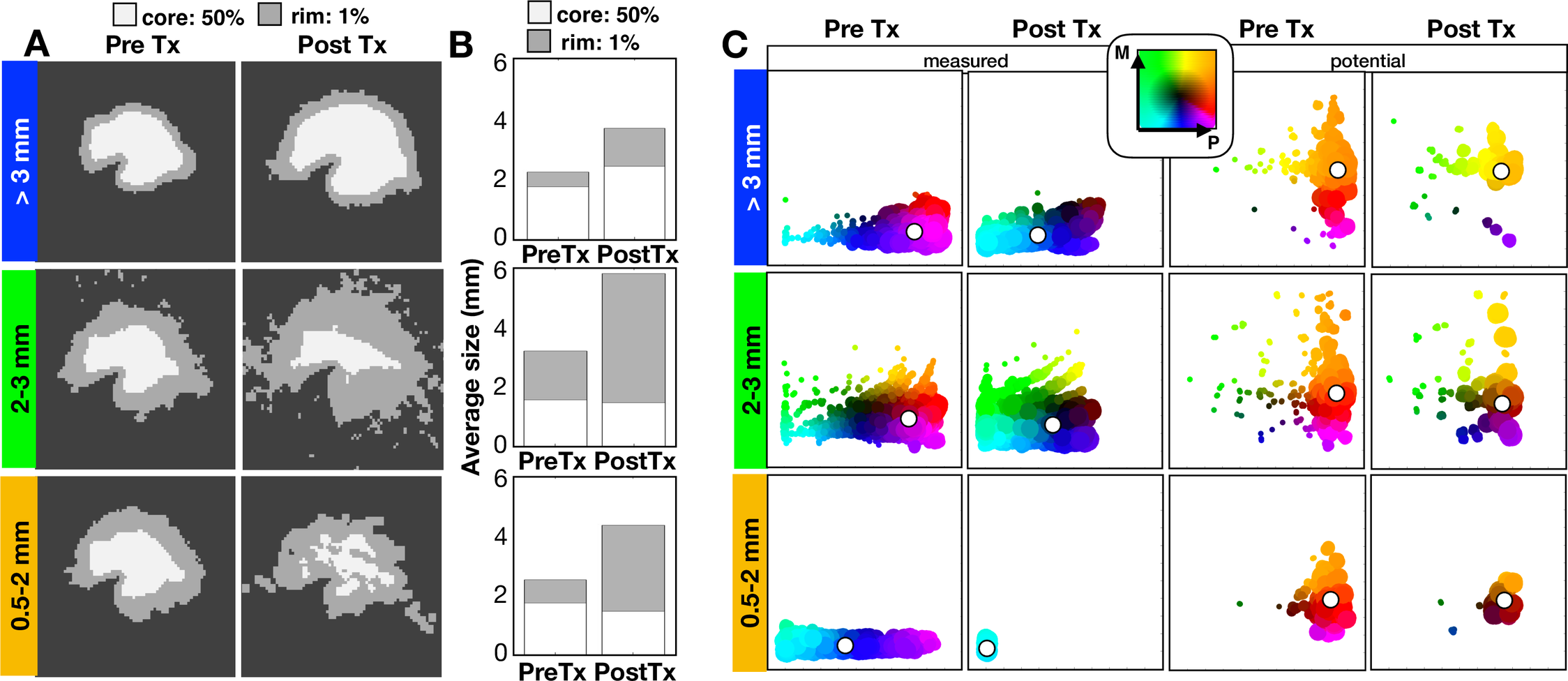

Supplement: S4 Fig — We compare the density distributions and single cell distributions of recurrent tumors of different diameters (>3mm, 2-3mm, and 0.5-2mm). A) The cellular density distributions define the core size (average diameter with a cell density of at least 50% of the carrying capacity) and the rim size (average diameter with a cell density of at least 1% of the carrying capacity). B) Stacked bar plot of average core diameter and rim diameter before and after treatment (over 10 runs). C) The measured and potential phenotype combinations for all non-quiescent cells within a single tumor are shown as a scatter plot. The color and location of each dot gives its proliferation rate and migration speed for each cell. The size of the circle is proportional to the number of cells with that phenotype combination, while a white dot marks the mean of the population. (TIF) [file pcbi.1007672.s009.tif]

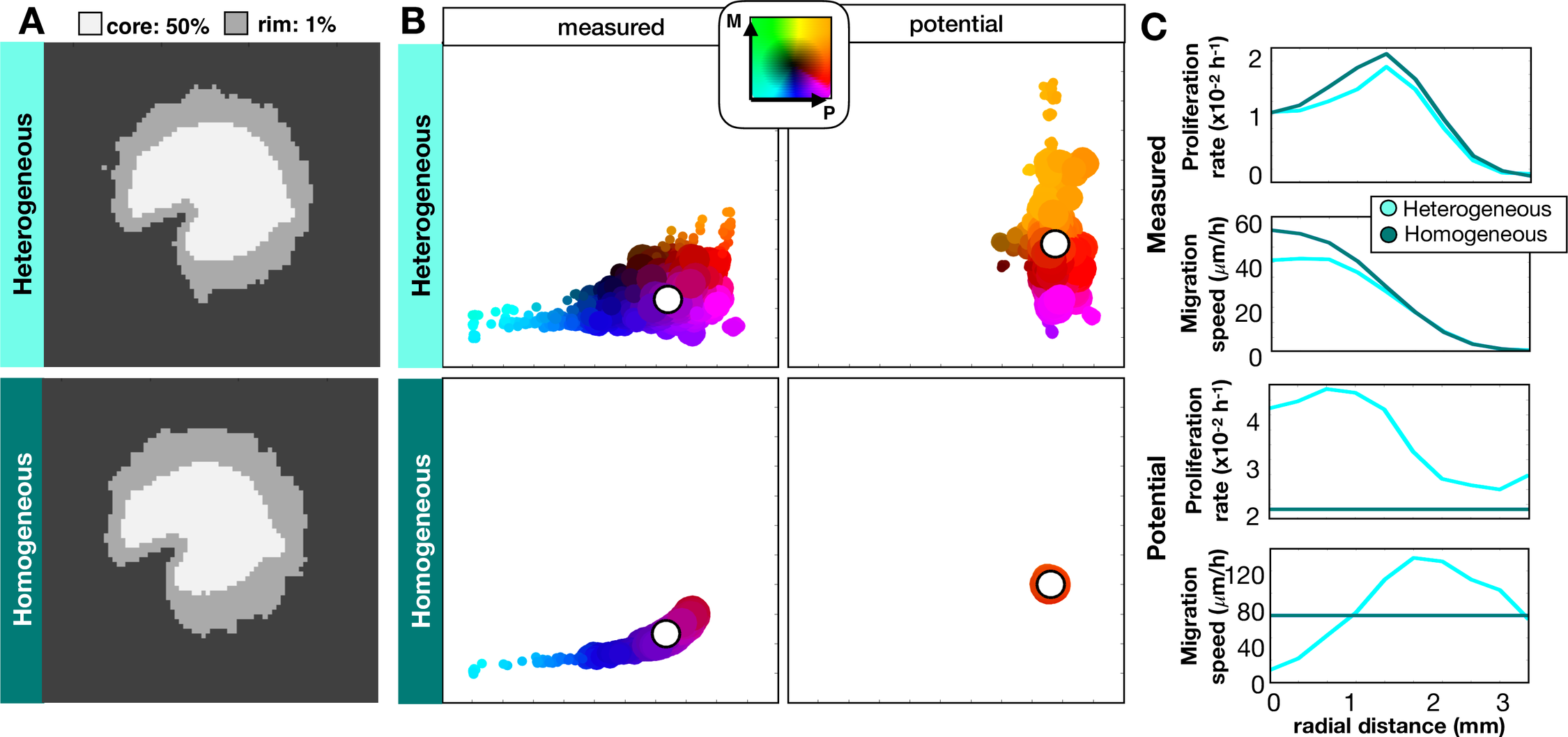

Supplement: S5 Fig — A) Tumor core and rim are determined from density distributions. The core is defined as having a cell density of at least 50% of the carrying capacity, while the rim is defined as having a cell density of at least 1% of the carrying capacity. For both tumors, the average core size was 1.9mm and average rim size was 0.6mm. B) The measured and potential phenotype combinations for all non-quiescent cells within a single tumor are shown as a scatter plot. The color and location of each dot gives its proliferation rate and migration speed for each cell. The size of the circle is proportional to the number of cells with that phenotype combination, while a white dot marks the mean of the population. C) Spatial phenotype distributions along the radius of the tumor at 17d. The average values over 10 runs are plotted for measured proliferation and migration rates and potential proliferation and migration rates. (TIF) [file pcbi.1007672.s010.tif]

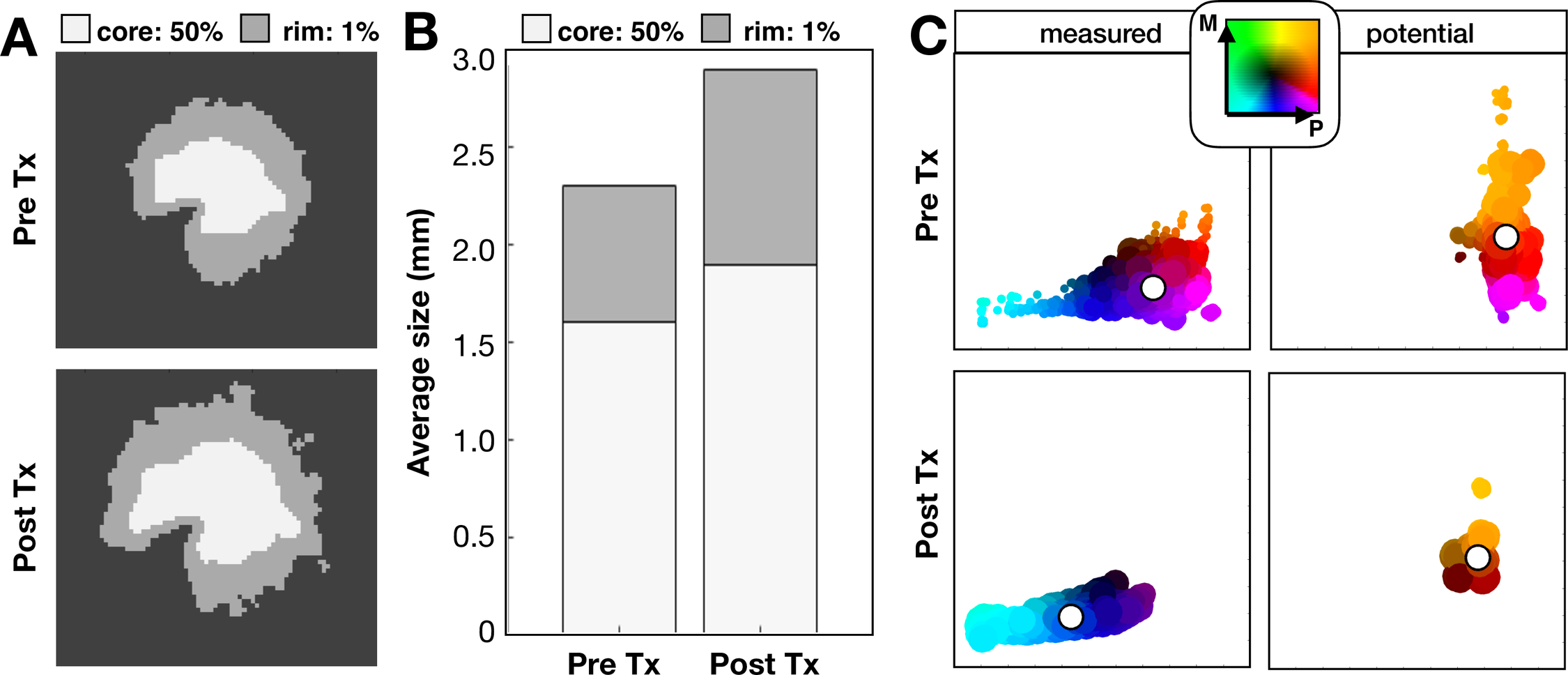

Supplement: S6 Fig — We compare the density distributions and single cell distributions of the recurrent heterogenous tumor before and after treatment. A) The cellular density distributions define the core size (average diameter with a cell density of at least 50% of the carrying capacity) and the rim size (average diameter with a cell density of at least 1% of the carrying capacity). B) The measured and potential phenotype combinations for all non-quiescent cells within a single tumor are shown as a scatter plot. The color and location of each dot gives its proliferation rate and migration speed for each cell. The size of the circle is proportional to the number of cells with that phenotype combination, while a white dot marks the mean of the population. C) Stacked bar plot of average core diameter and rim diameter before and after treatment (over 10 runs). (TIF) [file pcbi.1007672.s011.tif]

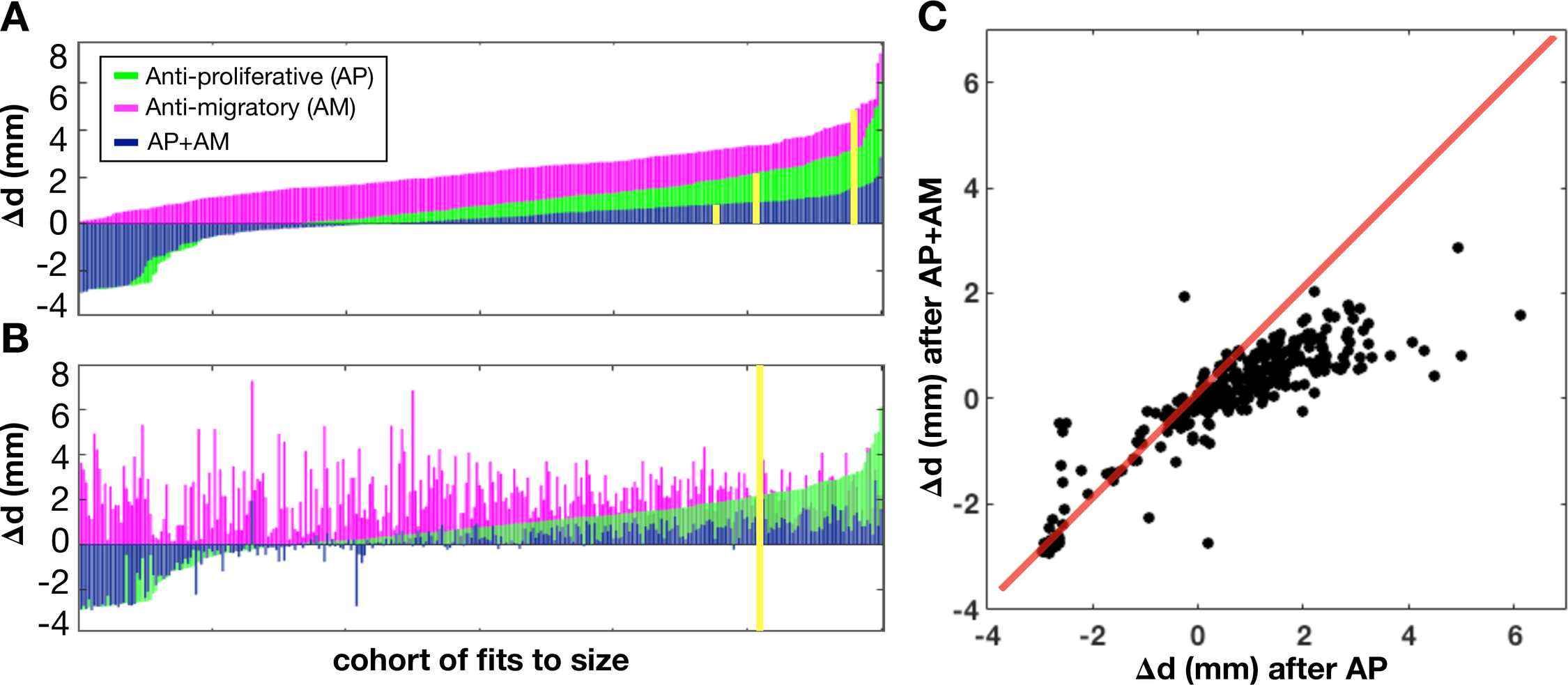

Supplement: S7 Fig — We show the distribution of response as A) a waterfall plot with each treatment sorted ranked from best to worst response and B) a waterfall plot for AP treatment sorted ranked from best to worst response but preserving the correlation of how each tumor responds to the other treatments. The yellow line shows the responses for the diffuse tumor from Fig 9. C) Comparison of the responses for AP treatment alone to AP+AM combination treatment. The red line shows where the response is the same for both treatments. (TIF) [file pcbi.1007672.s012.tif]

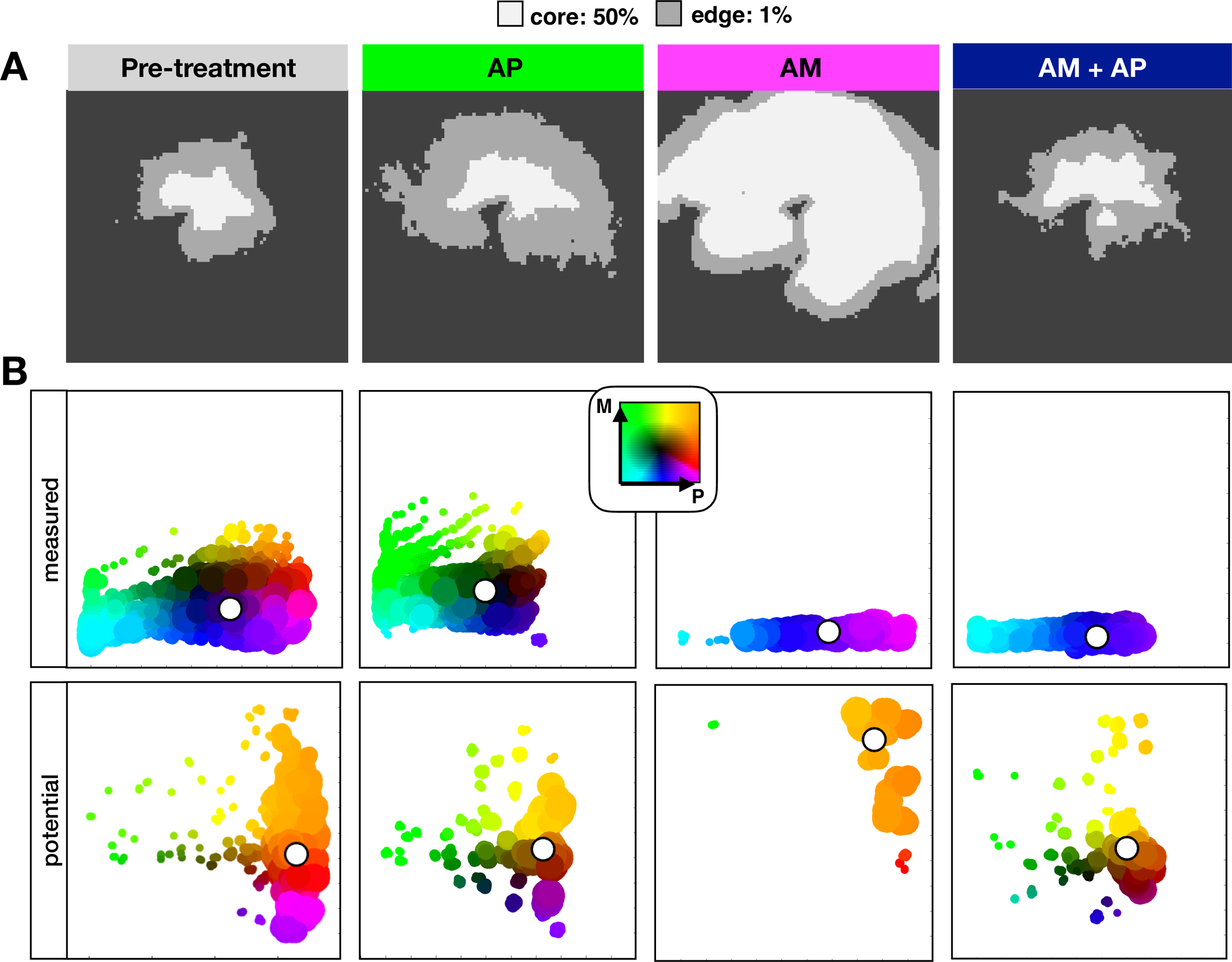

Supplement: S8 Fig — We compare the density distributions and single cell distributions of the pre-treatment (Pre Tx) and recurrent diffuse tumor after an anti-proliferative treatment (AP), an anti-migratory treatment (AM), and an anti-proliferative and anti-migratory combination (AP+AM). A) The cellular density distributions define the core size (average diameter with a cell density of at least 50% of the carrying capacity) and the rim size (average diameter with a cell density of at least 1% of the carrying capacity). B) The measured and potential phenotype combinations for all non-quiescent cells within a single tumor are shown as a scatter plot. The color and location of each dot gives its proliferation rate and migration speed for each cell. The size of the circle is proportional to the number of cells with that phenotype combination, while a white dot marks the mean of the population. (TIF) [file pcbi.1007672.s013.tif]

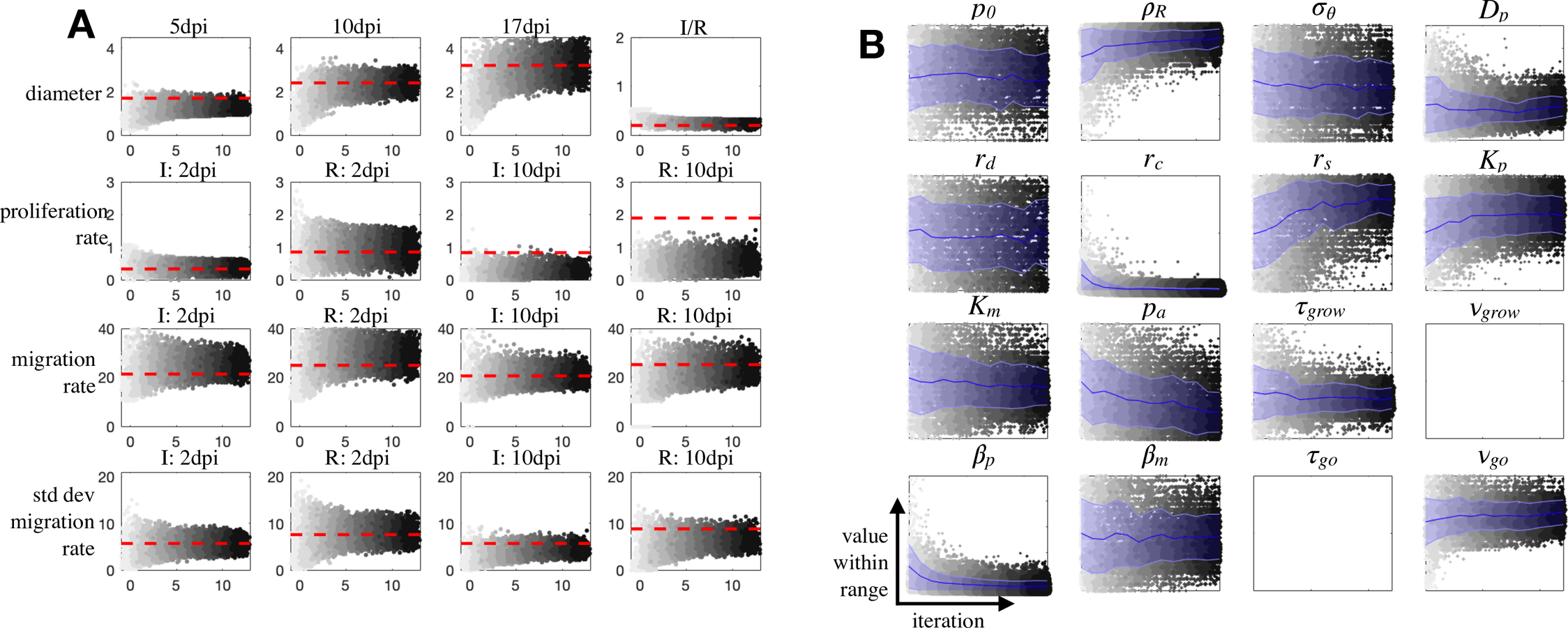

Supplement: S9 Fig — Values over iterations of the convergence are shown for A) metrics of top 300 fits using all data, and B) parameters from the top 300 fits using all data. Each iteration is shown starting at light gray and going to black for the final fit. The red dashed line for the metrics indicates the measured data values, while the blue lines and error bars show the mean and standard deviation over iterations for each parameter. For the “grow” population, τgrow was fit while νgrow = 0, and for the “go” population, τgrow = 200h, while νgrow was fit. (TIF) [file pcbi.1007672.s014.tif]

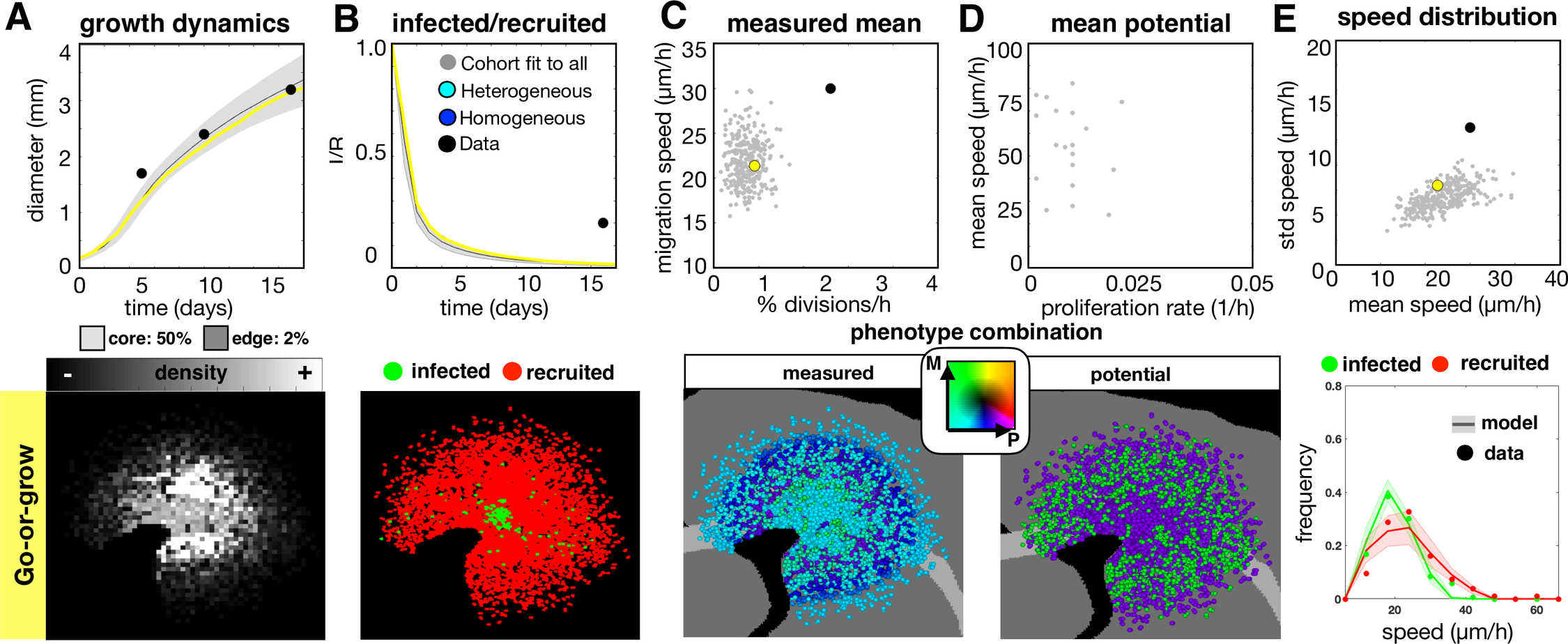

Supplement: S10 Fig — We show A) the growth dynamics, B) the infected/recruited ratio, C) the mean proliferation rate and mean migration speed combination, D) the potential trait combinations, and E) the mean and standard deviation of the speed distribution. Lower row, columns A-D show the spatial distributions at day 17 from which each metric is measured. The distribution of migration speeds from the single cell tracks is shown in the lower graph in column E). (TIF) [file pcbi.1007672.s015.tif]

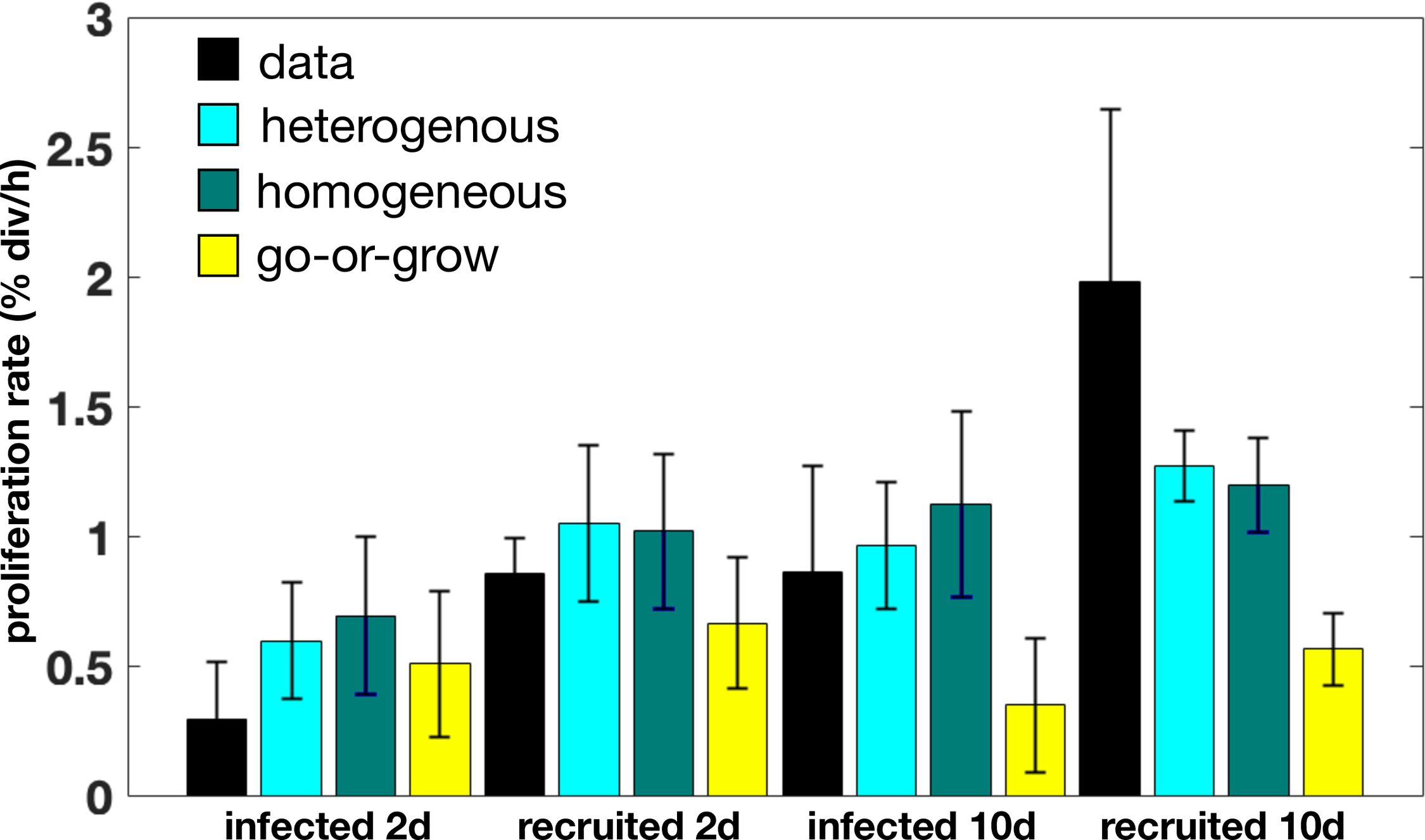

Supplement: S11 Fig — The error bar shows the resulting proliferation rate for the same best fit parameter set over 10 runs for each instance including: i) heterogeneous tumor: allowed heterogeneity in proliferation and migration, ii) homogeneous tumor: only environmental heterogeneity allowed, and iii) go-or-grow tumor: one cell type was fit to proliferation rate and allowed no migration, and one cell type was fit to migration speed with a slow proliferation rate (200h intermitotic time). (TIF) [file pcbi.1007672.s016.tif]
